# Adipose tissue area as a predictor for the efficacy of apatinib in platinum-resistant ovarian cancer: an exploratory imaging biomarker analysis of the AEROC trial

**DOI:** 10.1186/s12916-020-01733-4

**Published:** 2020-10-05

**Authors:** Xin Huang, Chuanbo Xie, Jie Tang, Wenzhuo He, Fan Yang, Wenfang Tian, Jundong Li, Qiuxia Yang, Jingxian Shen, Liangping Xia, Chunyan Lan

**Affiliations:** 1 Department of Gynecologic Oncology, Sun Yat-sen University Cancer Centre, State Key Laboratory of Oncology in South China, Collaborative Innovation Center for Cancer Medicine, 651 Dongfeng Road East, Guangzhou, 510060 Guangdong China; 2 Department of Cancer Prevention, Sun Yat-sen University Cancer Centre, State Key Laboratory of Oncology in South China, Collaborative Innovation Center for Cancer Medicine, Guangzhou, China; 3 Department of Gynecologic Oncology, Hunan Cancer Hospital, the Affiliated Cancer Hospital of Xiangya School of Medicine, Central South University, Changsha, China; 4 VIP Region, Sun Yat-sen University Cancer Centre, State Key Laboratory of Oncology in South China, Collaborative Innovation Center for Cancer Medicine, 651 Dongfeng Road East, Guangzhou, 510060 Guangdong China; 5 Department of Medical Imaging, Sun Yat-sen University Cancer Centre, State Key Laboratory of Oncology in South China, Collaborative Innovation Center for Cancer Medicine, Guangzhou, China

**Keywords:** Ovarian cancer, Visceral adipose tissue, Subcutaneous adipose tissue, Intermuscular adipose tissue, Vascular endothelial growth factor receptor

## Abstract

**Background:**

Vascular endothelial growth factor (VEGF)-targeted therapy is effective in patients with ovarian cancer. Whether adipose tissue (AT) could predict the efficacy of VEGF receptor (VEGFR) inhibitors in ovarian cancer is unknown. We aimed to evaluate the ability of distinct AT depots to predict the efficacy of apatinib, a VEGFR inhibitor, in recurrent ovarian cancers included in the AEROC trial.

**Methods:**

The AEROC was a single-arm phase 2 trial of apatinib and oral etoposide in patients with platinum-resistant or platinum-refractory ovarian cancer. Apatinib was administered continuously, and oral etoposide was administered every 21 days for a maximum of six cycles. This was a post hoc study based on the AEROC trial. Areas of visceral AT (VAT), subcutaneous AT (SAT), and intermuscular AT (IMAT) were measured using computed tomography scan at baseline to assess their association with the objective response rate, progression-free survival, and overall survival.

**Results:**

Of the 35 treated patients, 31 patients with at least one post-baseline efficacy assessment by computed tomography scan were included in this study. After adjusting for apatinib exposure, high VAT (odds ratio [OR], 0.16; 95% confidence interval [CI], 0.03–0.90, *P* = 0.037) and SAT (OR, 0.16; 95% CI, 0.03–0.87, *P* = 0.034) were significantly associated with a higher objective response rate. Further, decreased risks of disease progression and death were associated with high VAT (hazard ratio [HR], 0.39; 95% CI, 0.17–0.92, *P* = 0.031, and HR, 0.12; 95% CI, 0.04–0.40, *P* < 0.001, respectively), SAT (HR, 0.35; 95% CI, 0.15–0.83, *P* = 0.027, and HR, 0.24; 95% CI, 0.08–0.67, *P* = 0.007, respectively), and IMAT (HR, 0.20; 95% CI, 0.06–0.74, *P* = 0.016, and HR, 0.13; 95% CI, 0.03–0.62, *P* = 0.011, respectively).

**Conclusions:**

High areas of VAT, SAT, and IMAT were significantly associated with better outcomes in patients with platinum-resistant or platinum-refractory ovarian cancer who received VEGFR inhibitors. AT assessments may be valuable as patient-specific imaging biomarkers for predicting response to VEGFR inhibitors.

**Trial registration:**

ClinicalTrials.gov identifier: NCT02867956.

## Background

Ovarian cancer is the deadliest gynecologic malignancy and fifth most common cause of cancer mortality in women in developed countries [1]. Angiogenesis plays an important role in the natural history of ovarian cancer, promoting tumor growth and progression [2, 3]. The vascular endothelial growth factor (VEGF) signaling pathway is the most widely studied angiogenic pathway in ovarian cancer. The addition of anti-angiogenic drugs to chemotherapy, including VEGF receptor (VEGFR) tyrosine kinase inhibitors (TKIs), has shown clinical benefit in terms of progression-free survival (PFS) in patients with ovarian cancer, both in the upfront and recurrent settings [4, 5]. However, as not all patients benefit from anti-angiogenic therapy, identification of predictive biomarkers can help with the selection of patients responsive to this treatment. There is currently a lack of reliable and validated biomarkers for predicting the outcome of patients with ovarian cancer who receive VEGFR TKIs.

Obesity represents the excess accumulation of fat in adipose tissue (AT) and is known to increase the risk for quite diverse types of cancers [6, 7], including endometrial cancer, colorectal cancer, breast cancer, prostate cancer, and pancreatic cancer. It is also associated with higher risks of recurrence after treatment and death in many cancer types [8, 9]. The mechanisms linking excess AT and cancer development are not well understood, but obesity-associated chronic inflammation is widely accepted as an important factor in carcinogenesis [10, 11]. Moreover, it has been demonstrated that activated adipocytes produce multiple angiogenic factors, including VEGF, leptin, fibroblast growth factor-2 (FGF-2), tumor growth factor β (TGF-β), and placental growth factor (PGF), which stimulate neovascularization during AT expansion [12–14]. Indeed, adipogenesis is closely associated with angiogenesis [14]. Recently, several studies demonstrated that the areas of visceral AT (VAT) or subcutaneous AT (SAT) measured by computed tomography (CT) may predict the outcome of patients with metastatic colorectal cancer [15], renal cell cancer [16, 17], and melanoma [18], who were treated with VEGF-targeted therapy. However, these results were controversial.

Apatinib is a small molecule TKI that selectively inhibits VEGFR2. Previously, we reported the outcomes of a single-arm, phase 2 study (AEROC) on the efficacy of apatinib combined with oral etoposide in patients with platinum-resistant or platinum-refractory ovarian cancer [19]. The combination of apatinib and oral etoposide showed promising activity in this setting. In the current study, we evaluated the areas of VAT, SAT, and intermuscular AT (IMAT) using CT images that were collected prospectively in the AEROC trial. We analyzed the associations between AT areas and patient outcomes to evaluate whether distinct AT depots could predict the efficacy of apatinib in patients with platinum-resistant ovarian cancer.

## Methods

### Study design

The AEROC trial was a phase 2, single-arm, prospective study in patients with platinum-resistant or platinum-refractory ovarian cancer; the design and results have been previously reported [18]. In brief, the AEROC trial evaluated the efficacy and safety of apatinib and oral etoposide in patients with platinum-resistant or platinum-refractory ovarian cancer who had been treated with at least one line of platinum-based chemotherapy. The patients were treated with apatinib at an initial dose of 500 mg daily on a continuous basis and oral etoposide 50 mg once daily on days 1–14 of a 21-day cycle, for a maximum of six cycles. The primary endpoint was the objective response rate (ORR) using the Response Evaluation Criteria In Solid Tumors (RECIST) version 1.1. PFS and overall survival (OS) were the two main secondary endpoints.

As per protocol, dose modification, including dose interruptions and dose reductions, was performed based on the severity of toxicities according to NCI Common Terminology for Adverse Events (CTCAE), version 4.03 grading system. A maximum of two dose reductions were allowed for apatinib. The first dose reduction was from 500 mg once per day continuously to 500 mg and 250 mg taken on alternate days, and the additional dose reduction was to 250 mg once daily. If the apatinib dose was reduced, the dose could not be increased later. General guidelines for apatinib dose modification were presented in the protocol (Additional file 11: study protocol). The proportion of patients who had apatinib dose reduction and the reasons for apatinib dose reductions had been described previously [19].

The present study was a post hoc, secondary analysis of the AEROC trial. The primary objective was to evaluate the predictive value of VAT, SAT, and IMAT for the efficacy of apatinib-based treatment. The associations between areas of AT depots and ORR, updated PFS, and updated OS were investigated.

Informed consent was obtained from all patients. Ethical approval was obtained by the institutional review board of Sun Yat-sen University Cancer Centre. The procedures were in accordance with the ethical standards.

### CT analysis

**Fig. 1:**
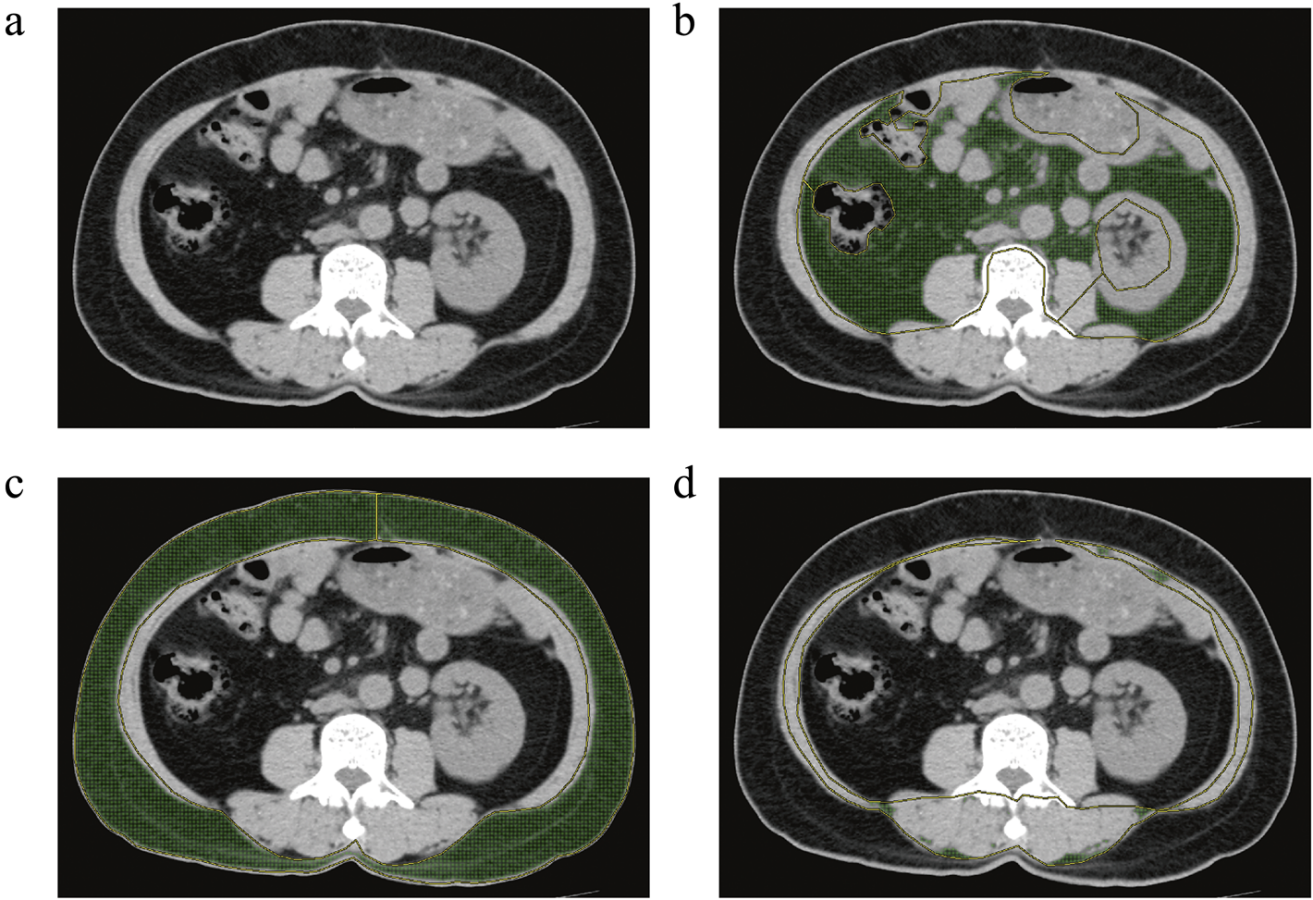
Representative axial CT images with respect to VAT, SAT, and IMAT. **a** The original CT image of AT and the segmentation of **b** VAT, **c** SAT, and **d** IMAT. CT, computed tomography; AT, adipose tissue; VAT, visceral AT; SAT, subcutaneous AT; IMAT, intermuscular AT

All patients underwent CT scans before the initiation of treatment for the purpose of efficacy assessment. CT examinations were performed on a clinical 64-slice CT scanner Discovery CT750 HD (GE Medical Systems; GE, Waukesha, USA), a Dual Source CT scanner (SOMATOM Force; SIEMENS, Erlangen, Germany), and iCT 256 (PHILIPS, Cleveland, USA). A single slice of each patient’s baseline CT image at the third lumbar vertebra (L3) was selected and analyzed by experienced radiologists blinded to patient information. The threshold was set between − 190 and − 30 Hounsfield units (HU) [20, 21], which is the attenuation range of AT on CT images. The VAT area was defined as intraabdominal fat bound by the parietal peritoneum or transversalis fascia, excluding the vertebral column and paraspinal muscles, with a density between − 150 and − 50 HU [20]. The SAT area was defined as the fat superficial to the abdominal and back muscles with attenuation ranging from − 190 to − 30 HU. The IMAT area was defined as the AT area visible between muscle groups and beneath the muscle fascia with attenuation ranging from − 190 to − 30 HU. The cross-sectional surface areas (cm^2^) of VAT, SAT, and IMAT were calculated automatically using the semi-automated GE Reformat post-processing tool (Centricity® Radiology RA1000; GE Medical Healthcare, Little Chalfont, UK). Representative axial CT images with respect to VAT, SAT, and IMAT are shown in Fig. 1.

### Statistical analyses

We used optimum stratification to define the optimal cutoffs for the areas of VAT, SAT, and IMAT [22]. Optimum stratification by SAS® macros solves the threshold value of continuous covariates with binary outcomes (SAS® macros %cutpoint) and time-to-event outcomes (SAS® macros %findcut) [22]. Therefore, the cutoffs for the areas of VAT, SAT, and IMAT to assess association with the ORR were determined using SAS® macros %cutpoint, and the cutoffs to assess association with PFS and OS were determined using SAS® macros %findcut. If more than one cutoff was suggested, the value that best distinguished patients with respect to ORR, PFS, and OS was selected. These cutoffs were then used to classify patients as having high and low VAT, SAT, and IMAT.

The distributions of patient characteristics according to the areas of VAT, SAT, and IMAT were compared using the chi-square or Fisher’s exact tests for categorical variables and independent *t* test for continuous variables. The relationships between the ORR and the areas of VAT, SAT, and IMAT were assessed using logistic regressions and adjusted for the dosage of apatinib. The Kaplan-Meier curves were used to display survival distributions, and the log-rank test was used to assess the difference between patients with high and low areas of AT depots. Hazard ratios (HRs) and corresponding 95% confidence intervals (CIs) for risk of disease progression and mortality associated with high and low areas of AT depots were estimated using Cox proportional hazards models. Multivariate Cox models were adjusted for the dosage of apatinib. All statistical analyses were performed using SAS version 9.4 (SAS Institute Inc., Cary, NC, USA). All tests were two-sided, and the significance level was set as 0.05.

## Results

### Patients

From August 10, 2016, to November 9, 2017, a total of 35 patients were enrolled in the AEROC trial. All 35 patients had baseline CT images. Four patients did not have post-baseline efficacy evaluation and were excluded from the current study. Thus, 31 patients with at least one post-baseline efficacy assessment by CT scan were included in this study. The data cutoff date used for the present analysis was February 7, 2019. The median follow-up was 13.8 months (range, 4.4–25.4 months). As of the data cutoff date, 2 patients were still receiving apatinib.

### Optimal cutoffs for the areas of VAT, SAT, and IMAT

An area of 55.53 cm^2^ was selected as the optimal cutoff for VAT associated with the ORR (Additional file 1: Table S1), PFS (Additional file 2: Fig. S1), and OS (Additional file 3: Fig. S2). The optimal cutoff for the area of SAT associated with ORR (Additional file 4: Table S2), PFS (Additional file 5: Fig. S3), and OS (Additional file 6: Fig. S4) was 129.28 cm^2^, and 3.28 cm^2^ was selected as the optimal cutoff for the area of IMAT associated with ORR (Additional file 7: Table S3), PFS (Additional file 8: Fig. S5), and OS (Additional file 9: Fig. S6). Patients with an area below these values were classified as having low VAT, SAT, and IMAT, respectively, and patients with an area above these values were classified as having high VAT, SAT, and IMAT, respectively. Representative axial CT images of two patients were provided (Additional file 10: Fig. S7): one with low VAT, low SAT, and low IMAT, and the other with high VAT, high SAT, and high IMAT.

### Patient characteristics according to areas of VAT, SAT, and IMAT

Patient characteristics according to VAT, SAT, and IMAT depots are summarized in Table 1. No significant differences in the majority of patient characteristics were observed between patients with high or low VAT, SAT, or IMAT. However, patients with high VAT (*P* = 0.049) and high SAT (*P* = 0.034) had significantly higher apatinib exposure (Table 1). Apatinib exposure was higher among patients with high IMAT than among patients with low IMAT, and the difference approached but did not reach significance (*P* = 0.096, Table 1).

**Table 1:** Patient characteristics according to VAT, SAT, and IMAT areas

Characteristic	All (*N* = 31)	VAT		*P* value	SAT		*P* value	IMAT		*P* value
		Low (*N* = 12)	High (*N* = 19)		Low (*N* = 12)	High (*N* = 19)		Low (*N* = 5)	High (*N* = 26)	
Age, *N* (%)				0.842			0.842			0.133*
< 60 years	20 (64.5)	8 (66.7)	12 (63.2)		8 (66.7)	12 (63.2)		5 (100)	15 (57.7)	
≥ 60 years	11 (35.5)	4 (33.3)	7 (36.8)		4 (33.3)	7 (36.8)		0 (0)	11 (42.3)	
Histology at diagnosis, *N* (%)				0.917*			0.917*			1.000*
High-grade serous carcinoma	24 (77.4)	9 (75.0)	15 (78.9)		9 (75.0)	15 (78.9)		5 (100)	19 (73.1)	
Low-grade serous carcinoma	1 (3.2)	0 (0)	1 (5.3)		0 (0)	1 (5.3)		0 (0)	1 (3.8)	
Endometrioid	2 (6.5)	1 (8.3)	1 (5.3)		1 (8.3)	1 (5.3)		0 (0)	2 (7.7)	
Clear cell	3 (9.7)	1 (8.3)	2 (10.5)		1 (8.3)	2 (10.5)		0 (0)	3 (11.5)	
Mucinous	1 (3.2)	1 (8.3)	0 (0)		1 (8.3)	0 (0)		0 (0)	1 (3.8)	
ECOG performance status, *N* (%)				0.644*			0.644*			0.656*
0	8 (25.8)	2 (16.7)	6 (31.6)		2 (16.7)	6 (31.6)		2 (40)	6 (23.1)	
1	22 (71.0)	10 (83.3)	12 (63.2)		10 (83.3)	12 (63.2)		3 (60)	19 (73.1)	
2	1 (3.2)	0 (0)	1 (5.3)		0 (0)	1 (5.3)		0 (0)	1 (3.8)	
Number of previous chemotherapy lines, *N* (%)				0.763			1.000			0.800*
1–2 lines	7 (22.6)	3 (25)	4 (21.1)		3 (25)	4 (21.1)		1 (20)	6 (23.1)	
3–6 lines	19 (61.3)	8 (66.7)	11 (57.9)		7 (58.3)	12 (63.2)		4 (80)	15 (57.7)	
> 6 lines	5 (16.1)	1 (8.3)	4 (21.1)		2 (16.7)	3 (15.8)		0 (0)	5 (19.2)	
Interval between last chemotherapy and disease progression, *N* (%)				0.676			0.676			0.562*
< 3 months	24 (77.4)	10 (83.3)	14 (73.7)		10 (83.3)	14 (73.7)		5 (100)	19 (73.1)	
≥ 3 months and < 6 months	7 (22.6)	2 (16.7)	5 (26.3)		2 (16.7)	5 (26.3)		0 (0)	7 (26.9)	
Dosage of apatinib, mean ± SD, g	69.86 ± 41.38	55.47 ± 24.84	78.95 ± 47.46	0.049	56.41 ± 24.02	78.36 ± 48.00	0.034	42.35 ± 16.23	75.15 ± 42.81	0.096

*Abbreviations*: *N* number, *VAT* visceral adipose tissue, *SAT* subcutaneous adipose tissue, *IMAT* intermuscular adipose tissue, *FIGO* International Federation of Gynecology and Obstetrics, *ECOG* Eastern Cooperative Oncology Group, *SD* standard deviation, *g* gram

*Fisher’s exact tests

### Association of AT depots with ORR

In univariate logistic regression, high VAT (*P* = 0.015) and high SAT (*P* = 0.015) were significantly associated with a higher ORR (Table 2). High IMAT was marginally associated with a higher ORR (*P* = 0.066). After adjusting for the dosage of apatinib, high VAT (odds ratio [OR], 0.16; 95% CI, 0.03–0.90, *P* = 0.037) and high SAT (OR, 0.16; 95% CI, 0.03–0.87, *P* = 0.034) were still associated with a higher ORR. However, the association between IMAT and response to apatinib was not significant when controlling for apatinib exposure (Table 2).

**Table 2:** Logistic regression analysis for AT associated with objective response rates

Variable	Univariate		Multivariate*	
	Odds ratio (95% CI)	*P* value	Odds ratio (95% CI)	*P* value
VAT				
High versus low	0.13 (0.03–0.68)	0.015	0.16 (0.03–0.90)	0.037
SAT				
High versus low	0.13 (0.03–0.68)	0.015	0.16 (0.03–0.87)	0.034
IMAT				
High versus low	0.11 (0.01–1.16)	0.066	0.18 (0.02–2.04)	0.166

*Abbreviations*: *AT* adipose tissue, *VAT* visceral adipose tissue, *SAT* subcutaneous adipose tissue, *IMAT* intermuscular adipose tissue, *CI* confidence interval

*Adjusted for apatinib exposure

### Association of AT depots with PFS

**Fig. 2:**
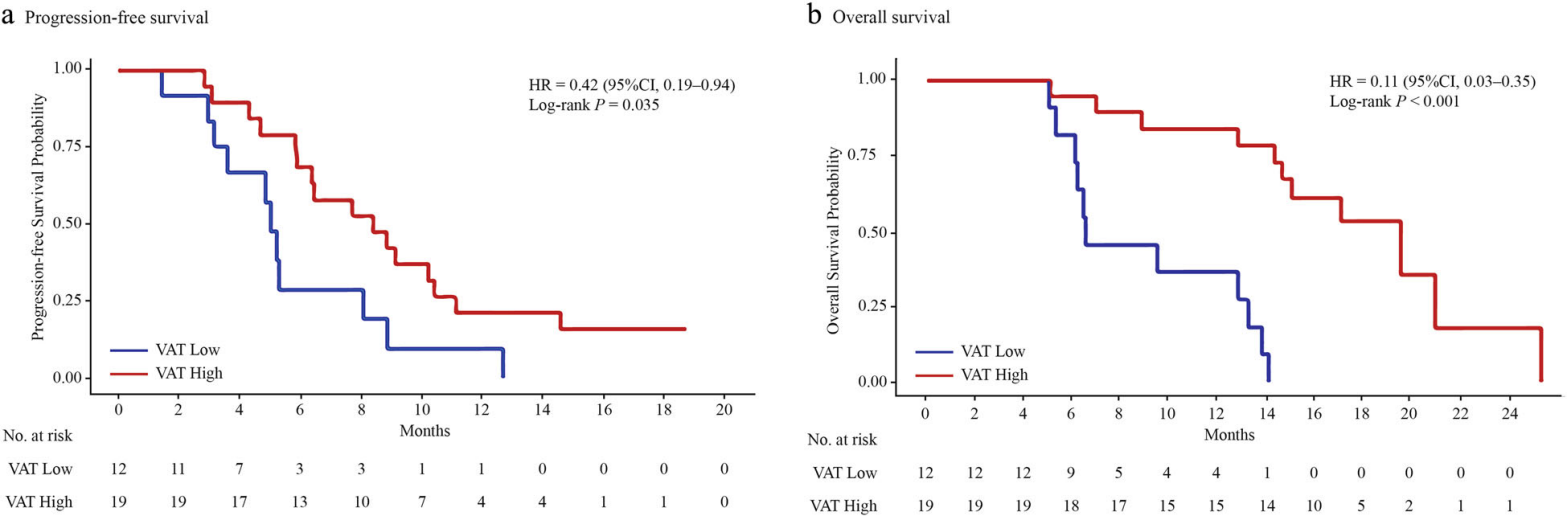
The Kaplan-Meier curves showing **a** progression-free survival and **b** overall survival in patients with low VAT and high VAT. VAT, visceral adipose tissue

**Fig. 3:**
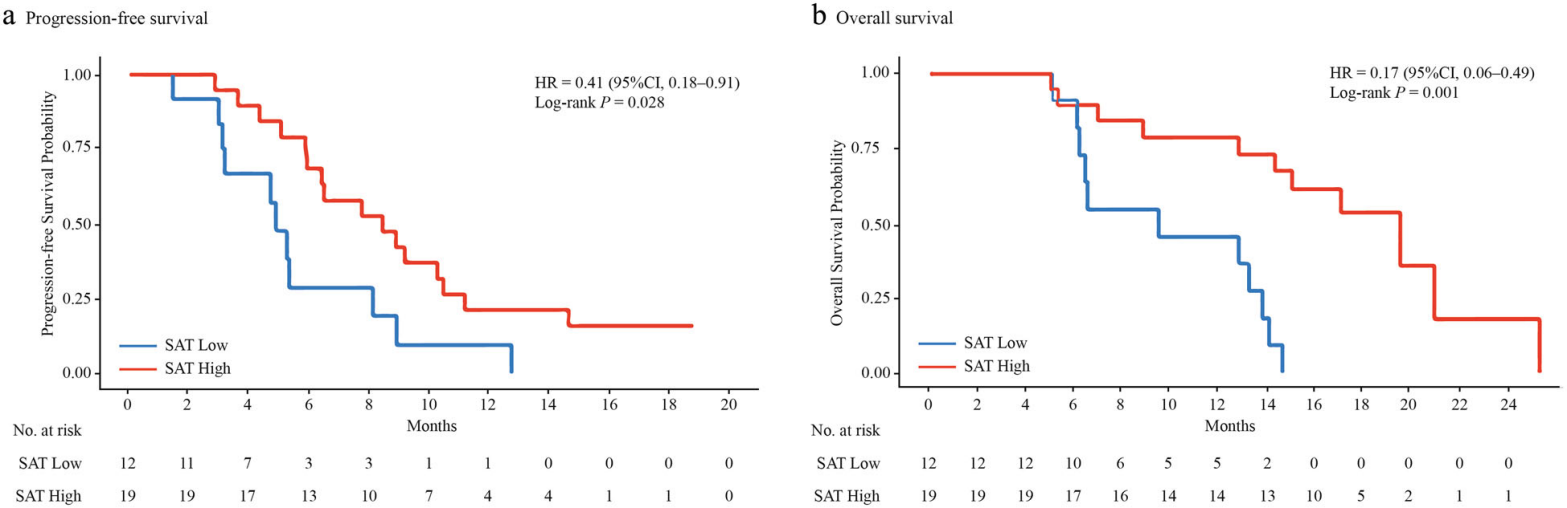
The Kaplan-Meier curves showing **a** progression-free survival and **b** overall survival in patients with low SAT and high SAT. SAT, subcutaneous adipose tissue

**Fig. 4:**
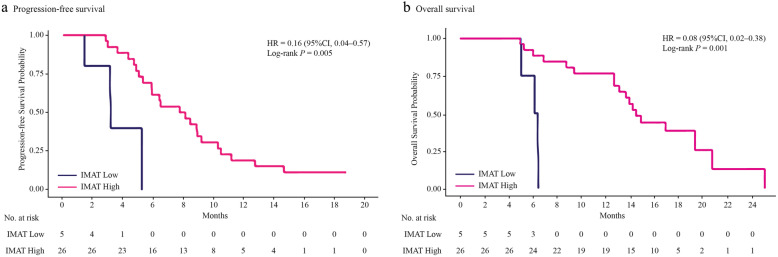
The Kaplan-Meier curves showing **a** progression-free survival and **b** overall survival in patients with low IMAT and high IMAT. IMAT, intermuscular adipose tissue

Univariate analysis demonstrated that patients with high VAT (Fig. 2a), high SAT (Fig. 3a), and high IMAT (Fig. 4a) were associated with a lower risk of disease progression than those with low VAT (*P* = 0.035), low SAT (*P* = 0.028), and low IMAT (*P* = 0.005), respectively. When adjusting for the dosage of apatinib (Table 3), high VAT was associated with a 61% (HR, 0.39; 95% CI, 0.17–0.92, *P* = 0.031) lower risk of disease progression compared with low VAT. High SAT and high IMAT were associated with a 65% and an 80% decreased risk of disease progression, respectively.

**Table 3:** Multivariate analysis for AT associated with progression-free survival and overall survival

Variable	Univariate			Multivariate*				
	Progression-free survival		Overall survival		Progression-free survival		Overall survival	
	HR (95% CI)	*P* value	HR (95% CI)	*P* value	HR (95% CI)	*P* value	HR (95% CI)	*P* value
VAT								
High versus low	0.42 (0.19–0.94)	0.035	0.11 (0.03–0.35)	< 0.001	0.39 (0.17–0.92)	0.031	0.12 (0.04–0.40)	< 0.001
SAT								
High versus low	0.41 (0.18–0.91)	0.028	0.17 (0.06–0.49)	0.001	0.35 (0.15–0.83)	0.027	0.24 (0.08–0.67)	0.007
IMAT								
High versus low	0.16 (0.04–0.57)	0.005	0.08 (0.02–0.38)	0.001	0.20 (0.06–0.74)	0.016	0.13 (0.03–0.62)	0.011

*Abbreviations*: *AT* adipose tissue, *VAT* visceral adipose tissue, *SAT* subcutaneous adipose tissue, *IMAT* intermuscular adipose tissue, *HR* hazard ratio, *CI* confidence interval

*Adjusted for apatinib exposure

### Association of AT depots with OS

Univariate analysis showed a lower risk for death among patients with high VAT (Fig. 2b), high SAT (Fig. 3b), and high IMAT (Fig. 4b) than patients with low VAT, low SAT, and low IMAT, respectively. With adjustment for the dosage of apatinib (Table 3), high VAT, high SAT, and high IMAT were associated with an 88% (HR, 0.12; 95% CI, 0.04–0.40, *P* < 0.001), a 76% (HR, 0.24; 95% CI, 0.08–0.67, *P* = 0.007), and an 87% (HR, 0.13; 95% CI, 0.03–0.62, *P* = 0.011) decreased risk of death, respectively.

## Discussion

This secondary analysis of the AEROC trial demonstrated an association between improved outcomes and high areas of VAT, SAT, and IMAT in patients with platinum-resistant or platinum-refractory ovarian cancer who received apatinib-based therapy. High areas of VAT and SAT predicted a better response to apatinib in this cohort. Moreover, PFS and OS were significantly superior in patients with high areas of VAT, SAT, and IMAT. Although apatinib exposure was higher among patients with high VAT, high SAT, and high IMAT, the areas of VAT, SAT, and IMAT still remained significant predictors for outcome of patients treated with apatinib after controlling for apatinib exposure. To our knowledge, this is the first time that the predictive value of AT area has been evaluated in patients with platinum-resistant ovarian cancer who received VEGFR inhibitors.

There are currently only a few studies on the predictive value of AT area in patients receiving VEGF-targeted treatment. In 64 patients with metastatic renal cell carcinoma treated with VEGF-targeted therapy, a high area of VAT was independently associated with shorter time to progression (HR, 3.07; 95% CI, 1.52–6.20; *P* = 0.002) and OS (HR, 6.26; 95% CI, 2.29–17.08; *P* < 0.001) [16]. In contrast, Steffens et al. [17] demonstrated that lower areas of VAT (HR, 3.26; 95% CI, 1.36–7.62; *P* = 0.006, and HR, 2.97; 95% CI, 1.36–6.47; *P* = 0.006, respectively) and SAT (HR, 2.66; 95% CI, 1.24–5.69; *P* = 0.012, and HR, 3.41; 95% CI, 1.61–7.25; *P* = 0.001, respectively) predicted shorter PFS and OS in patients in the same setting. The observations in the current study were in line with the work of Steffens et al. and gave further support that high areas of VAT and SAT predicted better outcomes in patients treated with VEGFR inhibitors.

Despite extensive efforts, much remains to be learned about the predictive value of AT in patients receiving VEGF-targeted treatment. There is increasing evidence that AT does not simply store energy but is also an essential endocrine organ, secreting a large number of inflammatory cytokines, which is favorable for tumor development [23, 24]. In particular, many cytokines produced by AT show angiogenic activities [13, 14]. This could explain why a large amount of AT is associated with high proangiogenic factor levels and therefore resistance to VEGF-targeted therapy [15, 16]. However, the crosstalk between angiogenesis and adipogenesis is complex [14, 25]. The consequences of modulation of angiogenic activity seem to be context-dependent. For instance, inhibition of VEGF-A in wild-type mice at the initial stages of high-fat food feeding causes aggravated systemic insulin resistance [25], which has been linked to several types of cancer [26–28]. However, the same blockade in mice with preexisting AT dysfunction had the opposite effect, with an improvement in insulin sensitivity and a decrease in inflammatory factors [25]. These findings may be a plausible explanation for the contradictory results regarding the predictive value of AT for VEGF-targeted treatment [15–18], and to some extent support our hypothesis that anti-angiogenic therapy in patients cancer with high adipose depots, who have preexisting AT dysfunction, is more likely to result in the reduction of inflammatory cytokines, which may favor a better outcome.

Until now, the optimal cutoff for specific adipose depots has not been well defined. Both the median value [15–17] and various tertiles [20, 29] have been used as cutoffs for AT area. Optimum stratification, which is widely used for identifying the threshold value of a continuous covariate by log-rank statistics testing [30], was used in this study to identify the cutoffs for different adipose depots. It should be noted that the patient population in the current study was small, and it is theoretically difficult to obtain statistically significant results. Surprisingly, we found that patients with different outcomes were well separated by the cutoffs identified by optimum stratification. This may be partly attributable to the optimum stratification method used in our study. Another plausible explanation might be that the areas of VAT, SAT, and IMAT are robust predictors of good outcomes in this patient population. However, further studies are warranted to validate these findings.

The adipose organ includes numerous discrete anatomical depots. Emerging evidence indicates that not all AT depots carry equivalent risk for metabolic abnormalities [31, 32]. However, the contribution of different AT depots to the response to VEGF-targeted therapy is unknown [16, 17]. Steffens et al. [17] identified the areas of both VAT and SAT as positive predictive biomarkers for patients with metastatic renal cell carcinoma treated with VEGF-targeted therapy. However, in the study of Ladoire et al. [16], no significant association was observed between areas of SAT and time to progression or OS in the same setting. In the current study, we demonstrated that all three adipose depots had a positive association with the outcome of patients receiving VEGFR inhibitors. To our knowledge, this is the first analysis to investigate the predictive value of IMAT for VEGF-targeted therapy. Interestingly, we further found that the largest reduction of risk of disease progression was observed in patients with high IMAT. Nevertheless, as IMAT is a novel and less well-studied AT depot, more data are needed to validate our findings.

We acknowledge that the patients in our study received oral etoposide as well which might to some extent contribute to the outcome of treatment. A growing body of evidence shows the existence of crosstalk between angiogenesis and adipogenesis [14, 33], providing the theoretical underpinnings of the association between AT area and outcome of treatment with apatinib. However, we could not show the difference in outcome for patients is purely attributable to the interaction of AT depots and apatinib.

### Limitations

Our study has several limitations. First, the AEROC trial was prospective but the current analysis was post hoc. The AEROC trial was not originally powered to evaluate the predictive value of AT for the efficacy of apatinib treatment. In addition, we did not internally validate our findings due to the small sample size. Therefore, we are not aware of the extent to which our findings could be replicated in this setting. Second, the AEROC trial was a single-arm trial with a relatively small sample size, and selection bias may affect the generalizability of the results.

## Conclusions

In this secondary analysis of the AEROC trial, high areas of VAT, SAT, and IMAT were significantly associated with better outcomes in patients with platinum-resistant ovarian cancer who received VEGFR inhibitor treatment. AT assessments may be valuable as patient-specific imaging biomarkers for predicting response to VEGFR inhibitor treatment and help individualize the treatment of patients with platinum-resistant ovarian cancer.

## Supplementary information

**Additional file 1: Table S1.** Best cutoffs for the areas of VAT associated with the objective response rate. Table S1 showed the performance of the proposed cutoffs selected by the SAS %cutpoint macro. The cutoff 33 cm^2^ achieved the highest total score. However, an area of 55.53 cm^2^ was selected instead of 33 cm^2^ as the optimal cutoff because it was not only significantly associated with objective response rate but also associated with progression-free survival and overall survival. VAT: visceral adipose tissue; CI, confidence interval.

**Additional file 2: Fig. S1.** Plot of cutoff selection for the area of VAT associated with progression-free survival. The x-axis represents the area of VAT and the y-axis shows the Wald *P* value. The horizontal dotted gray line indicates significance. Points above the line have a *P* > 0.05, and points below the line have a *P* < 0.05 and are suitable as cutoffs. VAT: visceral adipose tissue.

**Additional file 3: Fig. S2.** Plot of cutoff selection for the area of VAT associated with overall survival. The x-axis represents the area of VAT and the y-axis shows the Wald *P* value. The horizontal dotted gray line indicates significance. Points above the line have a *P* > 0.05, and points below the line have a *P* < 0.05 and are suitable as cutoffs. VAT: visceral adipose tissue.

**Additional file 4: Table S2.** Best cutoffs for the areas of SAT associated with the objective response rate. Table S2 showed the performance of the proposed cutoffs selected by the SAS %cutpoint macro. Although the area of 110 cm^2^ achieved the highest total score, the area of 129.28 cm^2^ was selected as the optimal cutoff because it was significantly associated with objective response rate as well as progression-free survival and overall survival. SAT: subcutaneous adipose tissue; CI: confidence interval.

**Additional file 5: Fig. S3.** Plot of cutoff selection for the area of SAT associated with progression-free survival. The x-axis represents the area of SAT and the y-axis shows the Wald *P* value. The horizontal dotted gray line indicates significance. Points above the line have a *P* > 0.05, and points below the line have a *P* < 0.05 and are suitable as cutoffs. SAT: subcutaneous adipose tissue.

**Additional file 6: Fig. S4.** Plot of cutoff selection for the area of SAT associated with overall survival. The x-axis represents the area of SAT and the y-axis shows the Wald *P* value. The horizontal dotted gray line indicates significance. Points above the line have a *P* > 0.05, and points below the line have a *P* < 0.05 and are suitable as cutoffs. SAT: subcutaneous adipose tissue.

**Additional file 7: Table S3.** Best cutoffs for the areas of IMAT associated with the objective response rate. Table S3 showed the performance of the proposed cutoffs selected by the SAS %cutpoint macro. The cutoff 5 cm2 had the highest total score. However, an area of 3.28 cm^2^ was selected instead of 5 cm^2^ as the optimal cutoff because it was not only significantly associated with objective response rate but also associated with progression-free survival and overall survival. IMAT: intermuscular adipose tissue; CI: confidence interval.

**Additional file 8: Fig. S5.** Plot of cutoff selection for the area of IMAT associated with progression-free survival. The x-axis represents the area of IMAT and the y-axis shows the Wald P value. The horizontal dotted gray line indicates significance. Points above the line have a P > 0.05, and points below the line have a P < 0.05 and are suitable as cutoffs. IMAT: intermuscular adipose tissue.

**Additional file 9: Fig. S6.** Plot of cutoff selection for the area of IMAT associated with overall survival. The x-axis represents the area of IMAT and the y-axis shows the Wald P value. The horizontal dotted gray line indicates significance. Points above the line have a P > 0.05, and points below the line have a P < 0.05 and are suitable for selection as cutoffs. IMAT: intermuscular adipose tissue.

**Additional file 10: Fig. S7.** Representative CT images for patients with high and low AT areas. (A) to (D): CT images for patients with high AT areas, (A) the original CT image of AT and the segmentation of (B) VAT, (C) SAT, and (D) IMAT. (E) to (H): CT images for patients with low AT areas, (E) the original CT image of AT and the segmentation of (F) VAT, (G) SAT, and (H) IMAT.

**Additional file 11**: Study protocol.

## Data Availability

The key raw data have been recorded at Research Data Deposit public platform (http://www.researchdata.org.cn) with number RDDA2019001126. The data are available from Research Data Deposit, but restrictions apply to the availability of these data, which were used under license for the current study, and so are not publicly available. Data are however available from the authors upon reasonable request and with permission of Research Data Deposit public platform.

## Abbreviations

VEGF: Vascular endothelial growth factor
VEGFR: VEGF receptor
TKI: Tyrosine kinase inhibitor
PFS: Progression-free survival
AT: Adipose tissue
FGF-2: Fibroblast growth factor-2
TGF-β: Tumor growth factor β
PGF: Placental growth factor
VAT: Visceral AT
SAT: Subcutaneous AT
CT: Computed tomography
IMAT: Intermuscular AT
ORR: Objective response rate
RECIST: Response Evaluation Criteria In Solid Tumors
OS: Overall survival
HU: Hounsfield units
HR: Hazard ratio
CI: Confidence interval
