# Supplementary material for: Adipose tissue area as a predictor for the efficacy of apatinib in platinum-resistant ovarian cancer: an exploratory imaging biomarker analysis of the AEROC trial
Source: BMC Med. 2020 Oct 5;18:267. doi: 10.1186/s12916-020-01733-4 (PMC7534164; doi:10.1186/s12916-020-01733-4)
Supplement: Supplementary file 1 — Additional file 1: Table S1. Best cutoffs for the areas of VAT associated with the objective response rate. Table S1 showed the performance of the proposed cutoffs selected by the SAS %cutpoint macro. The cutoff 33 cm2 achieved the highest total score. However, an area of 55.53 cm2 was selected instead of 33 cm2 as the optimal cutoff because it was not only significantly associated with objective response rate but also associated with progression-free survival and overall survival. VAT: visceral adipose tissue; CI, confidence interval. [file 12916_2020_1733_MOESM1_ESM.docx]

| **Proposed cutoffs** | ***P* value** | **Odds ratio** | **Lower CI limit** | **Upper CI limit** | **Total score** | ***P* value score** | **Odds rate score** |  |
| --- | --- | --- | --- | --- | --- | --- | --- | --- |
| 33 | .012 | 11.90 | 1.85 | 76.53 | 20 | 10 | 10 |  |
| 36 | .021 | 7.47 | 1.39 | 40.24 | 14 | 9 | 5 |  |
| 54 | .022 | 7.50 | 1.47 | 38.28 | 14 | 7 | 7 |  |
| 48 | .021 | 7.47 | 1.39 | 40.24 | 12 | 8 | 4 |  |
| 57 | .022 | 7.50 | 1.47 | 38.28 | 12 | 6 | 6 |  |
| 93 | .101 | 8.00 | 0.85 | 75.19 | 12 | 3 | 9 |  |
| 95 | .101 | 8.00 | 0.85 | 75.19 | 10 | 2 | 8 |  |
| 65 | .027 | 5.60 | 1.16 | 27.07 | 6 | 5 | 1 |  |
| 31 | .078 | 6.07 | 0.94 | 39.05 | 6 | 4 | 2 |  |
| 96 | .108 | 6.4 | 0.68 | 60.84 | 4 | 1 | 3 |  |
|  | | | | | | | | |
