# Supplementary material for: Adipose tissue area as a predictor for the efficacy of apatinib in platinum-resistant ovarian cancer: an exploratory imaging biomarker analysis of the AEROC trial
Source: BMC Med. 2020 Oct 5;18:267. doi: 10.1186/s12916-020-01733-4 (PMC7534164; doi:10.1186/s12916-020-01733-4)
Supplement: Supplementary file 2 — Additional file 2: Fig. S1. Plot of cutoff selection for the area of VAT associated with progression-free survival. The x-axis represents the area of VAT and the y-axis shows the Wald P value. The horizontal dotted gray line indicates significance. Points above the line have a P > 0.05, and points below the line have a P < 0.05 and are suitable as cutoffs. VAT: visceral adipose tissue. [file 12916_2020_1733_MOESM2_ESM.pdf]

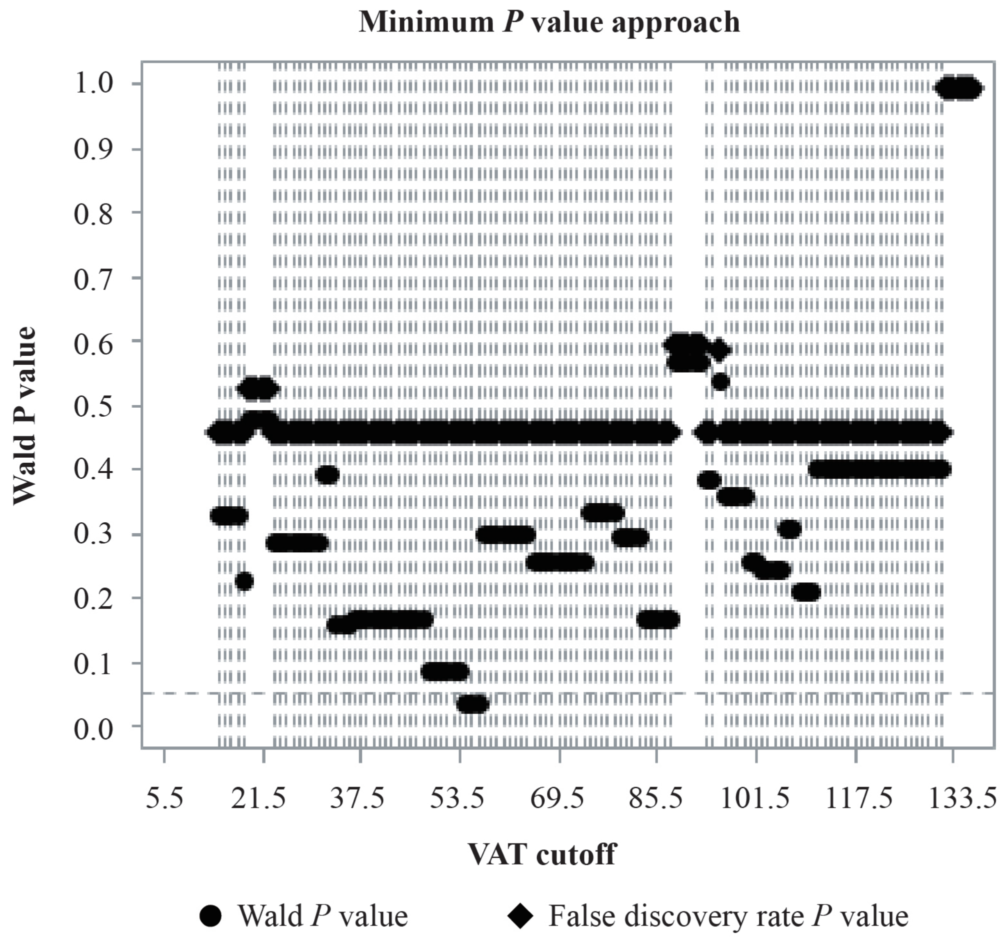

Cutoff	Cox model Wald *P* value		False discovery rate	
**VAT**	***P* value**	**Selected cutoff**	***P* value**	**Selected cutoff**
55.53	0.035	<====	0.454	<====
