# Supplementary material for: Adipose tissue area as a predictor for the efficacy of apatinib in platinum-resistant ovarian cancer: an exploratory imaging biomarker analysis of the AEROC trial
Source: BMC Med. 2020 Oct 5;18:267. doi: 10.1186/s12916-020-01733-4 (PMC7534164; doi:10.1186/s12916-020-01733-4)
Supplement: Supplementary file 4 — Additional file 4: Table S2. Best cutoffs for the areas of SAT associated with the objective response rate. Table S2 showed the performance of the proposed cutoffs selected by the SAS %cutpoint macro. Although the area of 110 cm2 achieved the highest total score, the area of 129.28 cm2 was selected as the optimal cutoff because it was significantly associated with objective response rate as well as progression-free survival and overall survival. SAT: subcutaneous adipose tissue; CI: confidence interval. [file 12916_2020_1733_MOESM4_ESM.docx]

| **Proposed cutoffs** | ***P* value** | **Odds ratio** | **Lower CI limit** | **Upper CI limit** | **Total score** | ***P* value score** | **Odds rate score** |
| --- | --- | --- | --- | --- | --- | --- | --- |
| 110 | .021 | 7.47 | 1.39 | 40.24 | 17 | 10 | 7 |
| 129 | .022 | 7.50 | 1.47 | 38.28 | 16 | 8 | 8 |
| 82 | .060 | 9.00 | 0.86 | 93.83 | 16 | 6 | 10 |
| 128 | .021 | 7.47 | 1.39 | 40.24 | 15 | 9 | 6 |
| 83 | .060 | 9.00 | 0.86 | 93.83 | 14 | 5 | 9 |
| 89 | .056 | 5.33 | 1.00 | 28.43 | 10 | 7 | 3 |
| 85 | .078 | 6.07 | 0.94 | 39.05 | 7 | 2 | 5 |
| 144 | .073 | 4.13 | 0.84 | 20.28 | 6 | 4 | 2 |
| 87 | .078 | 6.07 | 0.94 | 39.05 | 5 | 1 | 4 |
| 149 | .073 | 4.13 | 0.84 | 20.28 | 4 | 3 | 1 |
|  | | | | | | | |
