# Supplementary material for: Adipose tissue area as a predictor for the efficacy of apatinib in platinum-resistant ovarian cancer: an exploratory imaging biomarker analysis of the AEROC trial
Source: BMC Med. 2020 Oct 5;18:267. doi: 10.1186/s12916-020-01733-4 (PMC7534164; doi:10.1186/s12916-020-01733-4)
Supplement: Supplementary file 5 — Additional file 5: Fig. S3. Plot of cutoff selection for the area of SAT associated with progression-free survival. The x-axis represents the area of SAT and the y-axis shows the Wald P value. The horizontal dotted gray line indicates significance. Points above the line have a P > 0.05, and points below the line have a P < 0.05 and are suitable as cutoffs. SAT: subcutaneous adipose tissue. [file 12916_2020_1733_MOESM5_ESM.pdf]

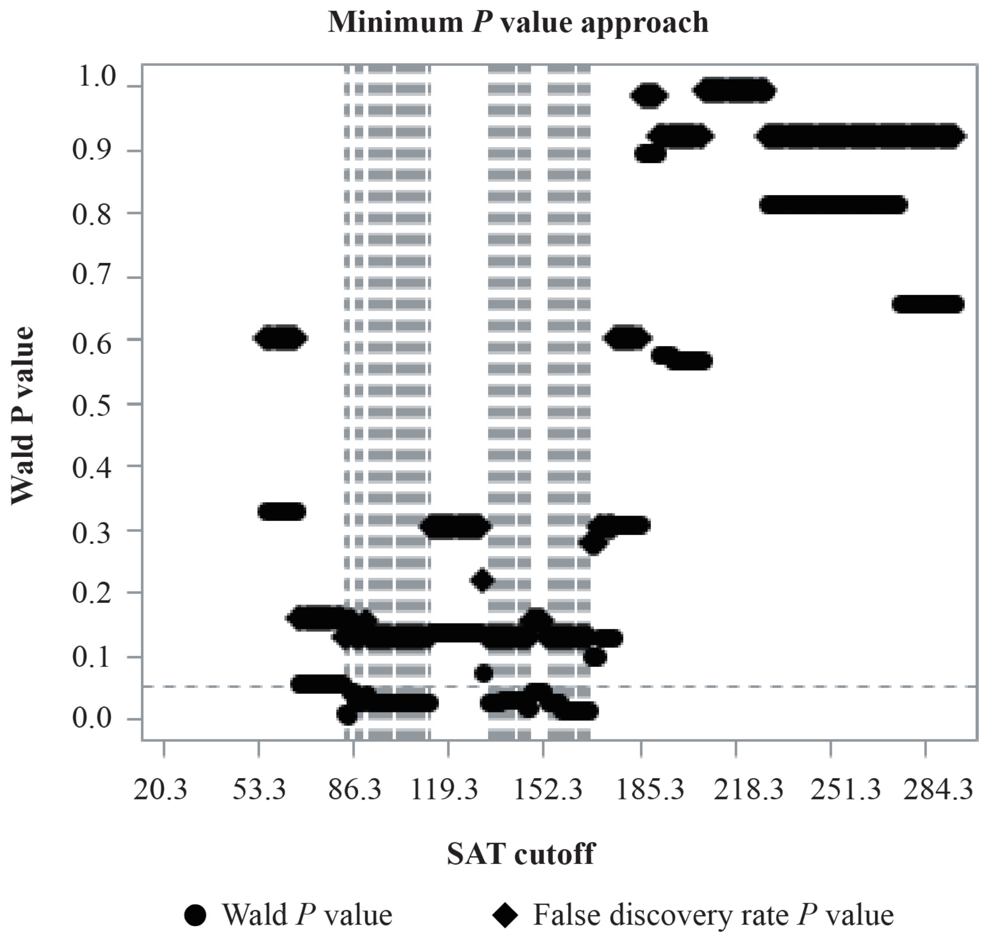

Cutoff	Cox model Wald *P* value		False discovery rate	
**SAT**	***P* value**	**Selected cutoff**	***P* value**	**Selected cutoff**
129.28	0.028	<====	0.128	<====
