# Supplementary material for: Adipose tissue area as a predictor for the efficacy of apatinib in platinum-resistant ovarian cancer: an exploratory imaging biomarker analysis of the AEROC trial
Source: BMC Med. 2020 Oct 5;18:267. doi: 10.1186/s12916-020-01733-4 (PMC7534164; doi:10.1186/s12916-020-01733-4)
Supplement: Supplementary file 7 — Additional file 7: Table S3. Best cutoffs for the areas of IMAT associated with the objective response rate. Table S3 showed the performance of the proposed cutoffs selected by the SAS %cutpoint macro. The cutoff 5 cm2 had the highest total score. However, an area of 3.28 cm2 was selected instead of 5 cm2 as the optimal cutoff because it was not only significantly associated with objective response rate but also associated with progression-free survival and overall survival. IMAT: intermuscular adipose tissue; CI: confidence interval. [file 12916_2020_1733_MOESM7_ESM.docx]

| **Proposed cutoffs** | ***P* value** | **Odds ratio** | **Lower CI limit** | **Upper CI limit** | **Total score** | ***P* value score** | **Odds rate score** |
| --- | --- | --- | --- | --- | --- | --- | --- |
| 5 | .127 | 3.75 | 0.77 | 18.21 | 12 | 7 | 5 |
| 3 | .270 | 6.00 | 0.54 | 66.17 | 11 | 5 | 6 |
| 4 | .253 | 2.68 | 0.55 | 13.16 | 10 | 6 | 4 |
| 10 | 1.000 | I | . | . | 8 | 1 | 7 |
| 7 | .433 | 2.31 | 0.38 | 13.96 | 7 | 4 | 3 |
| 6 | .552 | 1.56 | 0.36 | 6.69 | 5 | 3 | 2 |
| 9 | 1.000 | 1.29 | 0.10 | 16.04 | 3 | 2 | 1 |
