# Supplementary material for: Adipose tissue area as a predictor for the efficacy of apatinib in platinum-resistant ovarian cancer: an exploratory imaging biomarker analysis of the AEROC trial
Source: BMC Med. 2020 Oct 5;18:267. doi: 10.1186/s12916-020-01733-4 (PMC7534164; doi:10.1186/s12916-020-01733-4)
Supplement: Supplementary file 9 — Additional file 9: Fig. S6. Plot of cutoff selection for the area of IMAT associated with overall survival. The x-axis represents the area of IMAT and the y-axis shows the Wald P value. The horizontal dotted gray line indicates significance. Points above the line have a P > 0.05, and points below the line have a P < 0.05 and are suitable for selection as cutoffs. IMAT: intermuscular adipose tissue. [file 12916_2020_1733_MOESM9_ESM.pdf]

Minimum *P* value approach

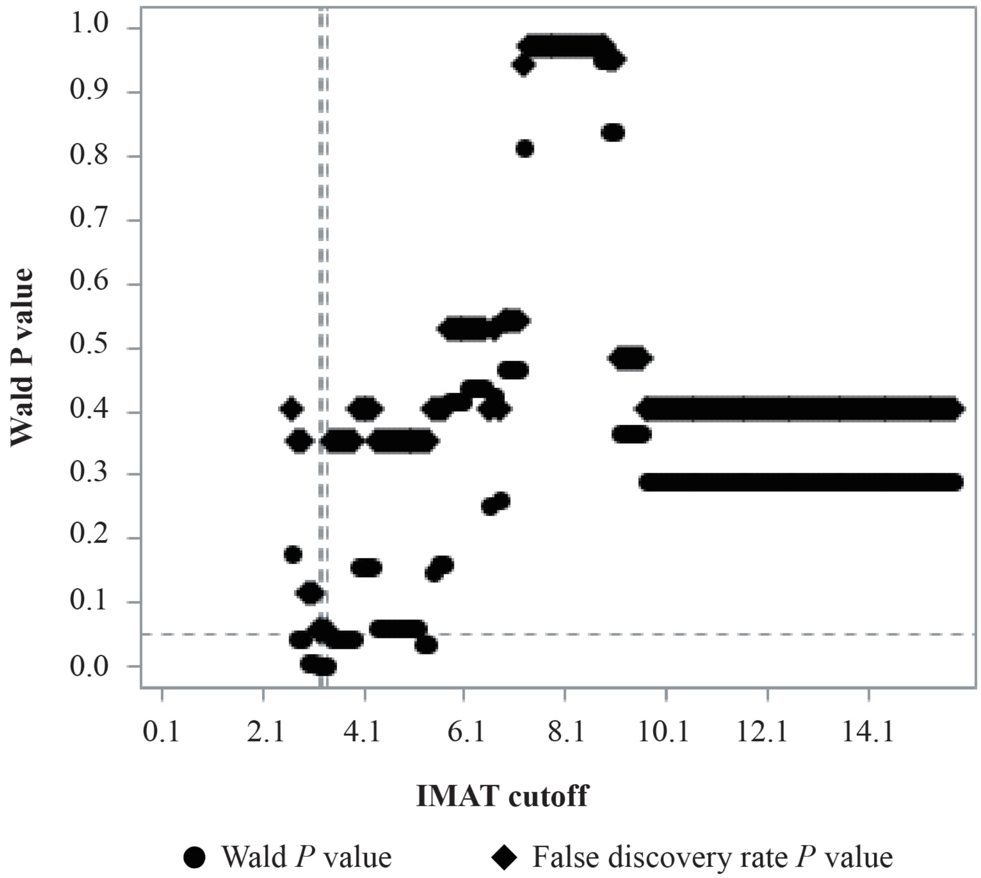


| Cutoff | Cox model Wald *P* value | | False discovery rate | |
|---|---|---|---|---|
| **IMAT** | ***P* value** | **Selected cutoff** | ***P* value** | **Selected cutoff** |
| 3.28 | 0.001 | <==== | 0.057 | <==== |
