# Supplementary figures and images for: Adipose tissue area as a predictor for the efficacy of apatinib in platinum-resistant ovarian cancer: an exploratory imaging biomarker analysis of the AEROC trial
Source: BMC Med. 2020 Oct 5;18:267. doi: 10.1186/s12916-020-01733-4 (PMC7534164; doi:10.1186/s12916-020-01733-4)

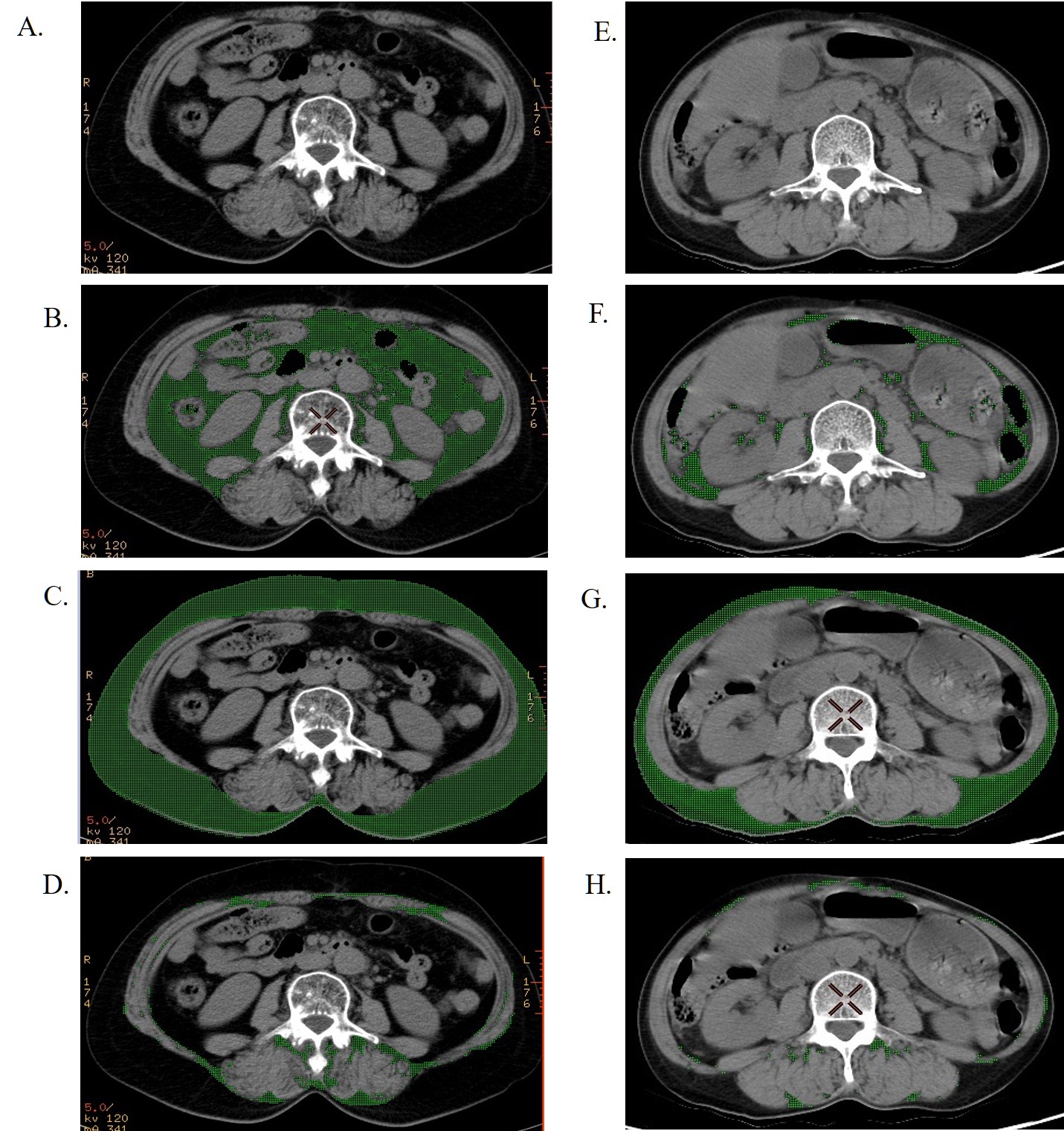

Supplement: Supplementary file 10 — Additional file 10: Fig. S7. Representative CT images for patients with high and low AT areas. (A) to (D): CT images for patients with high AT areas, (A) the original CT image of AT and the segmentation of (B) VAT, (C) SAT, and (D) IMAT. (E) to (H): CT images for patients with low AT areas, (E) the original CT image of AT and the segmentation of (F) VAT, (G) SAT, and (H) IMAT. [file 12916_2020_1733_MOESM10_ESM.jpg]
