# Supplementary material for: Adipose tissue area as a predictor for the efficacy of apatinib in platinum-resistant ovarian cancer: an exploratory imaging biomarker analysis of the AEROC trial
Source: BMC Med. 2020 Oct 5;18:267. doi: 10.1186/s12916-020-01733-4 (PMC7534164; doi:10.1186/s12916-020-01733-4)
Supplement: Supplementary file 11 — Additional file 11: Study protocol. [file 12916_2020_1733_MOESM11_ESM.doc]

**AEROC PROTOCOL**

**(Apatinib and Oral Etoposide in Platinum-Resistant or Platinum-refractory Ovarian Cancer)**

A Single-arm, Open-label, Phase 2 Study of Apatinib combined with Oral Etoposide in Patients with Platinum-resistant or Platinum-refractory Ovarian Cancer

Principal Investigator: Xin Huang, MD, Professor

Sun Yat-sen University Cancer Centre

Version： 6.0

Date：October, 2018

Contents

[1. Introductions 3](#__RefHeading___Toc14797259)

[2. Objectives 4](#__RefHeading___Toc14797260)

[2.1 Primary Objective 4](#__RefHeading___Toc14797261)

[2.2 Secondary Objectives 4](#__RefHeading___Toc14797262)

[2.3 Exploratory Objective 5](#__RefHeading___Toc14797263)

[3. Study Design and Sample Size 5](#__RefHeading___Toc14797264)

[3.1 Study Design 5](#__RefHeading___Toc14797265)

[3.2 Sample Size 5](#__RefHeading___Toc14797266)

[4. Subject Population 5](#__RefHeading___Toc14797267)

[4.1 Inclusion Criteria 5](#__RefHeading___Toc14797268)

[4.2 Exclusion Criteria 6](#__RefHeading___Toc14797269)

[5. Treatment and Administration 7](#__RefHeading___Toc14797270)

[6. Dose Modification 7](#__RefHeading___Toc14797271)

[6.1 Dose Interruptions 7](#__RefHeading___Toc14797272)

[6.2 Dose Reductions 7](#__RefHeading___Toc14797273)

[7. Study Procedures 9](#__RefHeading___Toc14797274)

[7.1 Screening Phase 9](#__RefHeading___Toc14797275)

[7.2 Treatment Phase 9](#__RefHeading___Toc14797276)

[7.3 Post-treatment Phase 10](#__RefHeading___Toc14797277)

[8. Efficacy 10](#__RefHeading___Toc14797278)

[8.1 Evaluations 10](#__RefHeading___Toc14797279)

[8.2 Endpoints 10](#__RefHeading___Toc14797280)

[9. Safety Evaluations 11](#__RefHeading___Toc14797281)

[9.1 Adverse Event 11](#__RefHeading___Toc14797282)

[9.2 Serious Adverse Event 12](#__RefHeading___Toc14797283)

[9.3 Attribution Definitions 12](#__RefHeading___Toc14797284)

[9.4 Safety Analyses 13](#__RefHeading___Toc14797285)

[10. Statistical Analysis Plan 13](#__RefHeading___Toc14797286)

[10.1 Analysis Populations 13](#__RefHeading___Toc14797287)

[10.1.1 Definitions of Study Populations 13](#__RefHeading___Toc14797288)

[10.1.2 Efficacy analysis populations 14](#__RefHeading___Toc14797289)

[10.1.3 Safety analysis population 14](#__RefHeading___Toc14797290)

[10.2 Methods of Statistical analysis 14](#__RefHeading___Toc14797291)

[10.2.1 Analysis of primary endpoint 14](#__RefHeading___Toc14797292)

[10.2.2 Analysis of secondary endpoints 14](#__RefHeading___Toc14797293)

[10.2.3 Safety analyses 15](#__RefHeading___Toc14797294)

[10.2.4 Analysis of exploratory endpoint 15](#__RefHeading___Toc14797295)

[10.3 Sample Size Determination 16](#__RefHeading___Toc14797296)

[11. Regulatory Ethics Compliance 16](#__RefHeading___Toc14797297)

[11.1 Investigator Responsibilities 16](#__RefHeading___Toc14797298)

[11.2 Informed Consent 16](#__RefHeading___Toc14797299)

[11.3 Compensation to Research Subjects 17](#__RefHeading___Toc14797300)

[11.4 Institutional Review Board (IRB) 17](#__RefHeading___Toc14797301)

[12. Administrative Requirements 17](#__RefHeading___Toc14797302)

[References 18](#__RefHeading___Toc14797303)

[Attachment 1: RECIST Guidelines 20](#__RefHeading___Toc14797304)

[Attachment 2: Performance status (ECOG scale) 36](#__RefHeading___Toc14797305)

# 1. Introductions

Ovarian cancer is the leading cause of death for patients with gynecologic malignancies. In most cases, the disease is diagnosed at an advanced stage and approximately 75% of patients will eventually experience disease recurrence. Almost all patients with recurrent disease ultimately develop platinum resistance, which results in death. For platinum-resistant ovarian cancer, a non-platinum-based agent (i.e., liposomal doxorubicin, topotecan, gemcitabine, and etoposide) is preferred. However, the overall response rates of these single-agent therapy remain in the 10-30%. Therefore, it is important to seek alternative agent that can improve the outcomes of patients with recurrent ovarian cancer.

Apatinib is a novel orally administered small-molecule tyrosine kinase inhibitor (TKI) that selectively binds to and inhibits vascular endothelial growth factor receptor 2 (VEGFR2). A series of studies have demonstrated that apatinib exhibits encouraging antitumor activities and tolerable toxicities in several malignant tumors. In a randomized, double-blind, placebo-controlled phase III study conducted by Li et al, apatinib improved the overall survival and progression-free survival in patients with chemotherapy-refractory advanced or metastatic gastric or gastroesophageal junction adenocarcinoma. In addition, a phase 2 study by Hu et al demonstrated that apatinib had objective efficacy in heavily pretreated, metastatic non-triple-negative breast cancer with manageable toxicity.

Angiogenesis is a hallmark process in cancer and is responsible for tumor spread and metastasis. It has been shown that anti-angiogenic therapy, including anti-VEGF antibodies and VEGFR/multi-receptor TKIs, represents an attractive therapeutic strategy for ovarian cancer. Moreover, increasing evidence has suggested that the combination of anti-angiogenic therapy and single-agent chemotherapy improves the outcome of platinum-resistant ovarian cancer. Among these single drugs, oral etoposide is one of the common treatments for patients with both platinum-resistant and platinum-sensitive disease.

On the basis of prior clinical experience, we carry out this trial to explore the efficacy and safety of the combination of apatinib and oral etoposide in patients with platinum-resistant or platinum-refractory ovarian cancer.

# 2. Objectives

## 2.1 Primary Objective

• Objective response rate (ORR)

Objective response rate defined as confirmed complete response or partial response under RECIST 1.1 criteria.

## 2.2 Secondary Objectives

• Progression-free survival (PFS)

Progression-free survival is defined as the time from registration to the earlier of death or disease progression. Patients alive without disease progression are censored at the date of last disease evaluation.

• Overall survival (OS)

Overall survival is defined as the time from registration until death from any cause (or date of censoring).

• Duration of Response (DOR)

Duration of response is defined as the interval between the date of the first documented response by RECIST to the date of first disease progression or death, whichever occurred earlier.

• Disease control rate (DCR)

Disease control rate is defined as the percentage of patients who achieved a complete response, a partial response or stable disease.

• Safety data

Frequency and severity of adverse effects as defined by CTCAE version 4.03

## 2.3 Exploratory Objective

• Imaging biomarkers for predictive modeling

To evaluate whether the areas of adipose tissue measured by computed tomography (CT) could predict the efficacy of apatinib.

# 3. Study Design and Sample Size

## 3.1 Study Design

This is a phase 2, single-arm, open-label study conducted at the Sun Yat-sen University Cancer Centre.

## 3.2 Sample Size

A Simon’s two-stage design is employed with a one-sided α-error of 5% and a power of 80%. The reported data indicated that the highest objective response rate of oral etoposide monotherapy in platinum-resistant ovarian cancer was 26.7%. We expected that the objective response rate for apatinib combined with etoposide would be 50%.

Under these assumptions, eight evaluable patients will be treated in stage one and more than 2 responses are required to continue to stage two. In the second stage, 27 more patients would be enrolled for a total sample size of 35. Overall, if a total of 14 responses or more are observed, the treatment regimen would be considered a success.

# 4. Subject Population

## 4.1 Inclusion Criteria

• Histologically or pathologically confirmed diagnosis of epithelial carcinoma of the ovary.

• Platinum-resistant ovarian cancer (defined as relapsing within 6 months after the last administration of platinum-based chemotherapy) OR platinum-refractory ovarian cancer (defined as progressing while on a platinum-based chemotherapy)

• At least treated with one line of platinum-based chemotherapy

• Female, age ≥18 years and ≤70 years, signed informed consent.

• Eastern Cooperative Oncology Group (ECOG) performance status 0-2

• Measurable disease according to Response Evaluation Criteria in Solid Tumors (RECIST) 1.1 version

• Patients must have a life expectancy of at least 3 months.

• Patients must have adequate organ function as defined by the following criteria:

• White blood cell count ≥ 3 × 10^9^/L, Absolute neutrophil count (ANC) (≥ 1.5× 10^9^/L), Hemoglobin of ≥ 80 g/L, Platelets ≥ 70 ×10^9^/L

• Total bilirubin ≤ 1 × upper limit of normal (ULN), AST and ALT ≤ 2 × ULN

• Serum creatinine ≤ 1 × ULN

## 4.2 Exclusion Criteria

• Had prior exposure to apatinib or has known allergies to any of the excipients.

• History of myocardial infarction, or unstable angina, or New York Heart Association (NYHA) Grade III-IV within 6 months prior to Day 1.

• Patients with QT interval prolongation

• Serious, non-healing wound, active ulcer, bowel obstruction

• History of abdominal fistula or gastrointestinal perforation within 28 days prior to Day 1

• Evidence of bleeding diathesis or coagulopathy

• Inadequately controlled hypertension

• Major surgical procedure within 28 days prior to Day 1

• Symptomatic central nervous system (CNS) metastasis

# 5. Treatment and Administration

• Apatinib combined with oral etoposide

• Apatinib, 500 mg, administered orally once daily

• Oral etoposide, 50 mg, administered on days 1 to 14. The administration of oral etoposide will be repeated every 3 weeks for a maximum of six cycles

Treatment was continued until disease progression, patient withdrawal, or unacceptable toxic effects.

# 6. Dose Modification

## 6.1 Dose Interruptions

Dose interruptions are required on the occurrence of grade 3 or worse toxicities. Treatment has to be delayed until recovery to ≤ grade 2 hematological or ≤ grade 1 nonhematological toxicities. Repeat dose interruptions are allowed for a maximum of 14 days on each occasion.

## 6.2 Dose Reductions

Dose reductions will be made on the basis of the worst drug-related toxicity. However, investigators may also reduce the dose of either or both study drugs for toxicity, as clinically warranted. Table 1 shows the dose levels that will be used when dose reductions are required. Patients who experience toxicity meeting the criteria cited in Table 2 will have a dose level reduction as specified in Table 1.

**Table 1. Dose level reductions —— Apatinib + Oral etoposide**

| Dose Level | Apatinib | Oral etoposide |
| --- | --- | --- |
| Starting level | 500mg/d，qd | 50mg/d，d1-d14 |
| Level-1 | 500 mg & 250 mg taken respectively every other day | 50mg/d，d1-d12 |
| Level-2 | 250mg/d，qd | 50mg/d，d1-d10 |

**Table 2. Dose reductions for toxicity**

| Toxicity | Worst CTC grade | | | Apatinib | Oral etoposide | |
| --- | --- | --- | --- | --- | --- | --- |
| Hematologic | | 1-2 | Not reduction | | | Not reduction |
|  |  | 3-4 | First occurrence: Not reduction | | | Decrease 1 level |
|  |  |  | Any occurrence after first occurrence: Decrease 1 level | | |  |
| Nonhematologic | |  |  | | |  |
| Hypertension,  Proteinuria，  Hand-foot syndrome | | 1-2 | Not reduction | | | Not reduction |
|  |  | 3 | First occurrence: Not reduction | | | Not reduction |
|  |  |  | Any occurrence after first occurrence: Decrease 1 level | | |  |
|  |  | 4 | Decrease 1 level | | | Not reduction |
| Others* | | 1-2 | Not reduction | | | Not reduction |
|  |  | 3 | First occurrence: Not reduction | | | Decrease 1 level |
|  |  |  | Any occurrence after first occurrence: Decrease 1 level | | |  |
|  |  | 4 | Decrease 1 level | | | Decrease 1 level |

*Other nonhematologic toxicities, e.g., elevated ALT/AST, elevated bilirubin, nausea, vomiting, and anorexia.

# 7. Study Procedures

## 7.1 Screening Phase

Screening procedures to be completed within 30 days before treatment:

• Signed informed consent

• Review of eligibility criteria

• Review of medical history and demographics

• Physical examination

• Vital signs

• Eastern Cooperative Oncology Group (ECOG) performance status

• Laboratory tests including hematology and serum chemistry, and urine routine.

• Tumor markers, including CA125, CA199, and HE4.

• ECG

• Tumor assessments, including radiographic imaging of the chest, abdomen and pelvis by computed tomography (CT) or magnetic resonance imaging (MRI).

## 7.2 Treatment Phase

During the treatment of oral etoposide combined with apatinib, patients had to come to outpatient clinic every 3 weeks. When six cycles of oral etoposide in combination with apatinib were completed, apatinib monotherapy was continued and patients had to come to outpatient clinic every one month.

• Laboratory tests including hematology and serum chemistry, and urine routine. Urinary protein quantity in 24 hours will be tested if urinary protein is 2+

• Tumor markers, including CA125, CA199, and HE4.

• Tumor assessment. Tumor response according to RECIST version 1.1 is assessed using computed tomography (CT) or magnetic resonance imaging (MRI) after two, four, and six cycles of oral etoposide, and subsequently every 2 months during apatinib monotherapy until confirmed disease progression.

In case of clinical progression, the date of progression will be defined as the date of the first imaging study that documents progression.

• Adverse events will be reported by the patients and be followed by the investigator until resolution or until a clinically stable endpoint is reached.

## 7.3 Post-treatment Phase

The subjects will continue on the schedule until: 1) radiographic disease progression, 2) the subject withdraws consent, 3) the subject begins subsequent anticancer therapy, or 4) the study is terminated. Once the subject discontinue the treatment, drug-related adverse events during treatment or within 30 days of last dose of the study should be recorded.

# 8. Efficacy

## 8.1 Evaluations

Measurable disease and the response criteria used in this protocol are defined in the RECIST guidelines (version 1.1) (attachment 1) and will be based on radiologic assessment only.

Appropriate radiological disease assessments (CT scans or MRI) will be performed before treatment, and should include, at a minimum, imaging of the chest, abdomen, and pelvis. Disease will be assessed using CT scans or MRI after two, four, and six cycles of oral etoposide, and subsequently every 2 months during apatinib monotherapy until confirmed disease progression.

## 8.2 Endpoints

**Primary endpoints**

• Objective response rate (ORR), is defined as confirmed complete response or partial response under RECIST 1.1 criteria.

**Secondary endpoints**

• Progression-free survival (PFS), is defined as the time from registration to the earlier of death or disease progression.

• Overall survival (OS), is defined as the time from registration until death from any cause (or date of censoring).

• Duration of Response (DOR), is defined as the interval between the date of the first documented response by RECIST to the date of first disease progression or death, whichever occurred earlier.

• Disease control rate (DCR), is defined as the percentage of patients who achieved a complete response, a partial response or stable disease.

• Safety will be described according to the frequency of adverse effects that occur in the treatment.

**Exploratory endpoint**

• The association between the areas of adipose tissue measured by CT scans and efficacy

# 9. Safety Evaluations

## 9.1 Adverse Event

An adverse events is any untoward medical occurrence in a clinical study subject administered a medicinal product. An adverse event does not necessarily have a causal relationship with the treatment. An adverse event can therefore be any unfavorable and unintended sign, symptom, or disease temporally associated with the use of a medicinal product, whether or not related to that medicinal product.

This includes any occurrence that is new in onset or aggravated in severity or frequency from the baseline condition, or abnormal results of diagnostic procedures, including laboratory test abnormalities.

## 9.2 Serious Adverse Event

A Serious Adverse Event (SAE) is defined by FDA and NCI as any adverse drug event (experience) occurring at any dose:

• Results in death

• Is life-threatening

• Requires inpatient hospitalization or prolongation of existing hospitalization

• Results in persistent or significant disability/incapacity

• Is a congenital anomaly/birth defect

• Important Medical Event (IME) that may not result in death, be life threatening, or require hospitalization may be considered a serious adverse drug experience when, based upon medical judgment, they may jeopardize the patient or subject and may require medical or surgical intervention to prevent one of the outcomes listed in this definition.

All SAEs occurring during the study must be reported to Institutional Review Board (IRB) with 24 hours of their knowledge of the event. The initial and follow-up reports of a SAE should be made.

## 9.3 Attribution Definitions

An adverse event is considered associated with the use of the drug if the attribution is possible, probable, or very likely by the definitions.

• **Not Related**

An adverse event that is not related to the use of the drug.

**• Doubtful**

An adverse event for which an alternative explanation is more likely, e.g., concomitant drug(s), concomitant disease(s), or the relationship in time suggests that a causal relationship is unlikely.

**• Possible**

An adverse event that might be due to the use of the drug. An alternative explanation, e.g., concomitant drug(s), concomitant disease(s), is inconclusive. The relationship in time is reasonable; therefore, the causal relationship cannot be excluded.

**• Probable**

An adverse event that might be due to the use of the drug. The relationship in time is suggestive (e.g., confirmed by dechallenge). An alternative explanation is less likely, e.g., concomitant drug(s), concomitant disease(s).

**• Very likely**

An adverse event that is listed as a possible adverse reaction and cannot be reasonably explained by an alternative explanation, e.g., concomitant drug(s), concomitant disease(s). The relationship in time is very suggestive.

## 9.4 Safety Analyses

All subjects who have received at least 1 dose of study drug will be included in the safety analysis. All the adverse events should be recorded in CRF and will be graded according to the NCI-CTCAE, Version 4.03. Serious adverse and deaths will be listed. All adverse events resulting in discontinuation of study treatment, dose modification, the interruption of dosing, or a delay in treatment with the study drug will be noted.

# 10. Statistical Analysis Plan

## 10.1 Analysis Populations

### 10.1.1 Definitions of Study Populations

In this study the following three populations will be defined for the analysis.

• Intent-to-Treat (ITT) Population: The ITT population is defined as all patients who are enrolled in the trial.

• Per Protocol (PP) Population: The per protocol population (PP) defines as a subset of the patients who meet all of the trial criteria and are compliant with the protocol and absence of any major protocol violations.

• Safety Population: The safety population includes the enrolled patients who received at least one dose of study drug.

### 10.1.2 Efficacy analysis populations

The efficacy analyses will be based on the ITT population and PP population.

### 10.1.3 Safety analysis population

The safety population will be used for the analysis of all safety parameters.

## 10.2 Methods of Statistical analysis

### 10.2.1 Analysis of primary endpoint

Objective response rate (ORR) is defined as the proportion of patients who have achieved either CR or PR as defined as RECIST (version 1.1). All other patients will be considered non-responders in the analysis. The objective response rate will be summarized (i.e., number of patients (%)) and the 95% Pearson-Clopper confidence interval for the objective response rate will be provided.

### 10.2.2 Analysis of secondary endpoints

Progression-free survival (PFS) is defined as the time from registration to the earlier of death or disease progression. Patients alive without disease progression are censored at the date of last disease evaluation.

Overall survival (OS), is defined as the time from registration until death from any cause (or date of censoring).

Duration of Response (DOR) is defined as the time from the first CR or PR to the first event of disease progression or death.

The Kaplan-Meier method will be used to estimate the distribution of PFS, OS and DOR. The number of events, subjects censored, the estimate of medians and 95%CIs for the medians will be presented.

Disease Control Rate (DCR) is defined as the percentage of patients who achieved CR, PR or SD. The disease control rate will be summarized (i.e., number of patients (%)) and the 95% Pearson-Clopper confidence interval for the disease control rate will be provided.

### 10.2.3 Safety analyses

All patients who received at least one dose of study drug will be included in the safety analysis. The safety parameters to be evaluated are the incidence, intensity, and type of adverse events, and clinical laboratory results. All adverse events and abnormal laboratory variables will be assessed according to the NCI-CTCAE, Version 4.03 grading system. For each adverse event, the percentage of patients who had at least 1 occurrence of the given event will be summarized. Serious adverse events and deaths will be listed. All adverse events resulting in discontinuation of study treatment, dose modification, the interruption of dosing will be summarized.

Descriptive statistics will be used to summarise ECOG performance status.

### 10.2.4 Analysis of exploratory endpoint

The association between adipose tissue (AT) depots, including visceral AT (VAT), subcutaneous AT (SAT), and intermuscular AT (IMAT), and the efficacy will be evaluated.

The areas of VAT, SAT, and IMAT will be measured by CT scan. The relationships between the ORR and the areas of VAT, SAT, and IMAT will be assessed using logistic regressions. The associations between PFS and OS and the areas of VAT, SAT, and IMAT will be assessed using Kaplan-Meier method and log-rank test.

## 10.3 Sample Size Determination

A Simon’s two-stage design is employed with a one-sided α-error of 5% and a power of 80%. The reported data indicated that the highest objective response rate of oral etoposide monotherapy in platinum-resistant ovarian cancer was 26.7%. We expected that the objective response rate for apatinib combined with etoposide would be 50%.

Under these assumptions, eight evaluable patients will be treated in stage one and more than 2 responses are required to continue to stage two. In the second stage, 27 more patients would be enrolled for a total sample size of 35. Overall, if a total of 14 responses or more are observed, the treatment regimen would be considered a success.

# 11. Regulatory Ethics Compliance

## 11.1 Investigator Responsibilities

The investigator is responsible for ensuring that the clinical study is performed in accordance with the protocol, current ICH guideline on Good Clinical Practice (GCP), and applicable regulatory and country-specific requirements.

## 11.2 Informed Consent

Each subject must give written consent according to local requirements after the nature of the study has been fully explained. The informed consent should be in accordance with principles that originated in the Declaration of Helsinki, current ICH and GCP guidelines, applicable regulatory requirements.

Before enrollment in the study, the investigator must explain to potential subjects the aims, methods, reasonably anticipated benefits, and potential hazards of the study, any discomfort participation in the study may entail. Subjects will be informed that their participation is voluntary and that they may withdraw consent to participate at any time. They will be informed that choosing not to participate will not affect the care the subject will receive for the treatment. The subject will be given the opportunity to ask questions. After the explanation and before entry into the study, consent should be recorded by the subject’s personally dated signature. After having obtained the consent, a copy of the ICF must be given to the subject.

## 11.3 Compensation to Research Subjects

A subject is entitled to compensation if injury or death is due to adverse effect of investigational products. Compensation must be consistent with the laws, regulations, and guidelines of the region in which the study is conducted.

## 11.4 Institutional Review Board (IRB)

Before the start of the study, the investigator will provide the IRB with current and complete copies of the documents, which include, but are not limited to, final protocol, informed consent, investigators’ curriculum vitae, information regarding funding, and other potential conflicts of interest. The study will be undertaken only after the IRB has given full approval of all the documents. All the protocol amendments must be submitted to the IRB for review and approval before implementation of the changes.

# 12. Administrative Requirements

The investigators should performed the following aspects, which include, but are not limited to, protocol amendments, regulatory documentation, case report form completion, record retention, monitoring, and data quality control.

# References

1. Mutch DG, Orlando M, Goss T, et al: Randomized phase III trial of gemcitabine compared with pegylated liposomal doxorubicin in patients with platinum-resistant ovarian cancer. J Clin Oncol 2007; 25: 2811–18.
2. Gordon AN, Tonda M, Sun S, et al. Long-term survival advantage for women treated with pegylated liposomal doxorubicin compared with topotecan in a phase 3 randomized study of recurrent and refractory epithelial ovarian cancer. Gynecol Oncol 2004; 95: 1–8.
3. Li J, Qin S, Xu J, et al. Apatinib for chemotherapy-refractory advanced metastatic gastric cancer: results from a randomized, placebo-controlled, parallel-arm, phase II trial. J Clin Oncol. 2013; 31:3219–25.
4. Li J, Qin S, Xu J, et al. Randomized, Double-Blind, Placebo-Controlled Phase III Trial of Apatinib in Patients With Chemotherapy-Refractory Advanced or Metastatic Adenocarcinoma of the Stomach or Gastroesophageal Junction. J Clin Oncol. 2016; 34:1448–54.
5. Hu X, Cao J, Hu W, et al. Multicenter phase II study of apatinib in non-triple-negative metastatic breast cancer. BMC Cancer. 2014; 14:820.
6. Perren TJ, Swart AM, Pfisterer J, et al. A phase 3 trial of bevacizumab in ovarian cancer. N Engl J Med 2011; 365: 2484–96.
7. Pujade-Lauraine E, Hilpert F, Weber B, et al: Bevacizumab combined with chemotherapy for platinum-resistant recurrent ovarian cancer: The AURELIA open-label randomized phase III trial. J Clin Oncol 2014; 32:1302–08.
8. Pignata S, Lorusso D, Scambia G, et al. Pazopanib plus weekly paclitaxel versus weekly paclitaxel alone for platinum-resistant or platinum-refractory advanced ovarian cancer (MITO 11): a randomised, open-label, phase 2 trial. Lancet Oncol 2015; 16: 561–68.
9. Rose PG, Blessing JA, Mayer AR, et al. Prolonged oral etoposide as second-line therapy for platinum-resistant and platinum-sensitive ovarian carcinoma: a Gynecologic Oncology Group study. J Clin Oncol. 1998; 16:405–10.

# Attachment 1: RECIST Guidelines

The following information was extracted from Section 3, Seciton 4, and Appendix I of the New response evaluation criteria in solid tumours: revised RECIST guideline (version 1.1).

**Measurability of tumour at baseline**

**Definitions**

At baseline, tumour lesions/lymph nodes will be categorized measurable or non-measurable as follows:

Measurable

*Tumour lesions:*

Must be accurately measured in at least one dimension (longest diameter in the plane of measurement is to be recorded) with a minimum size of:

• 10 mm by CT scan (CT scan slice thickness no greater than 5 mm; see Appendix II on imaging guidance).

• 10 mm caliper measurement by clinical exam (lesions which cannot be accurately measured with calipers should be recorded as non-measurable).

• 20 mm by chest X-ray

*Malignant lymph nodes:*

To be considered pathologically enlarged and measurable, a lymph node must be ≥15 mm in short axis when assessed by CT scan (CT scan slice thickness recommended to be no greater than 5 mm). At baseline and in follow-up, only the short axis will be measured and followed (see Schwartz et al. in this Special Issue). See also notes below on ‘Baseline documentation of target and non-target lesions’ for information on lymph node measurement.

Non-measurable

All other lesions, including small lesions (longest diameter<10 mm or pathological lymph nodes with ≥10 to <15 mm short axis) as well as truly non-measurable lesions. Lesions considered truly non-measurable include: leptomeningeal disease, ascites, pleural or pericardial effusion, inflammatory breast disease, lymphangitic involvement of skin or lung, abdominal masses/abdominal organomegaly identified by physical exam that is not measurable by reproducible imaging techniques.

**Special considerations regarding lesion measurability**

Bone lesions, cystic lesions, and lesions previously treated with local therapy require particular comment:

*Bone lesions:*

• Bone scan, PET scan or plain films are not considered adequate imaging techniques to measure bone lesions. However, these techniques can be used to confirm the presence or disappearance of bone lesions.

• Lytic bone lesions or mixed lytic-blastic lesions, with identifiable soft tissue components, that can be evaluated by cross sectional imaging techniques such as CT or MRI can be considered as measurable lesions if the soft tissue component meets the definition of measurability described above.

• Blastic bone lesions are non-measurable.

*Cystic lesions:*

• Lesions that meet the criteria for radiographically defined simple cysts should not be considered as malignant lesions (neither measurable nor non-measurable) since they are, by definition, simple cysts.

• ‘Cystic lesions’ thought to represent cystic metastases can be considered as measurable lesions, if they meet the definition of measurability described above. However, if noncystic lesions are present in the same patient, these are preferred for selection as target lesions.

*Lesions with prior local treatment:*

• Tumour lesions situated in a previously irradiated area, or in an area subjected to other loco-regional therapy, are usually not considered measurable unless there has been demonstrated progression in the lesion. Study protocols should detail the conditions under which such lesions would be considered measurable.

**Specifications by methods of measurements**

*Measurement of lesions*

All measurements should be recorded in metric notation, using calipers if clinically assessed. All baseline evaluations should be performed as close as possible to the treatmentstart and never more than 4 weeks before the beginning of the treatment.

*Method of assessment*

The same method of assessment and the same technique should be used to characterise each identified and reported lesion at baseline and during follow-up. Imaging based evaluation should always be done rather than clinical examination unless the lesion(s) being followed cannot be imaged but are assessable by clinical exam.

*Clinical lesions:* Clinical lesions will only be considered measurable when they are superficial and P10 mm diameter as assessed using calipers (e.g. skin nodules). For the case of skin lesions, documentation by colour photography including a ruler to estimate the size of the lesion is suggested. As noted above, when lesions can be evaluated by both clinical exam and imaging, imaging evaluation should be undertaken since it is more objective and may also be reviewed at the end of the study.

*Chest X-ray:* Chest CT is preferred over chest X-ray, particularly when progression is an important endpoint, since CT is more sensitive than X-ray, particularly in identifying new lesions. However, lesions on chest X-ray may be considered measurable if they are clearly defined and surrounded by aerated lung. See Appendix II for more details.

*CT, MRI:* CT is the best currently available and reproducible method to measure lesions selected for response assessment. This guideline has defined measurability of lesions on CT scan based on the assumption that CT slice thickness is 5 mm or less. As is described in Appendix II, when CT scans have slice thickness greater than 5 mm, the minimum size for a measurable lesion should be twice the slice thickness. MRI is also acceptable in certain situations (e.g. for body scans). More details concerning the use of both CT and MRI for assessment of objective tumour response evaluation are provided in Appendix II.

*Ultrasound:* Ultrasound is not useful in assessment of lesion size and should not be used as a method of measurement. Ultrasound examinations cannot be reproduced in their entirety for independent review at a later date and, because they are operator dependent, it cannot be guaranteed that the same technique and measurements will be taken from one assessment to the next (described in greater detail in Appendix II). If new lesions are identified by ultrasound in the course of the study, confirmation by CT or MRI is advised. If there is concern about radiation exposure at CT, MRI may be used instead of CT in selected instances.

*Endoscopy, laparoscopy:* The utilisation of these techniques for objective tumour evaluation is not advised. However, they can be useful to confirm complete pathological response when biopsies are obtained or to determine relapse in trials where recurrence following complete response or surgical resection is an endpoint.

*Tumour markers:* Tumour markers alone cannot be used to assess objective tumour response. If markers are initially above the upper normal limit, however, they must normalise for a patient to be considered in complete response. Because tumour markers are disease specific, instructions for their measurement should be incorporated into protocols on a disease specific basis. Specific guidelines for both CA-125 response (in recurrent ovarian cancer) and PSA response (in recurrent prostate cancer), have been published. In addition, the Gynecologic Cancer Intergroup has developed CA125 progression criteria which are to be integrated with objective tumour assessment for use in first-line trials in ovarian cancer.

*Cytology, histology:* These techniques can be used to differentiate between PR and CR in rare cases if required by protocol (for example, residual lesions in tumour types such as germ cell tumours, where known residual benign tumours can remain). When effusions are known to be a potential adverse effect of treatment (e.g. with certain taxane compounds or angiogenesis inhibitors), the cytological confirmation of the neoplastic origin of any effusion that appears or worsens during treatment can be considered if the measurable tumour has met criteria for response or stable disease in order to differentiate between response (or stable disease) and progressive disease.

**Tumour response evaluation**

**Assessment of overall tumour burden and measurable disease**

To assess objective response or future progression, it is necessary to estimate the overall tumour burden at baseline and use this as a comparator for subsequent measurements. Only patients with measurable disease at baseline should be included in protocols where objective tumour response is the primary endpoint. Measurable disease is defined by the presence of at least one measurable lesion (as detailed above in Section 3). In studies where the primary endpoint is tumour progression (either time to progression or proportion with progression at a fixed date), the protocol must specify if entry is restricted to those with measurable disease or whether patients having non-measurable disease only are also eligible.

**Baseline documentation of ‘target’ and ‘non-target’ lesions**

When more than one measurable lesion is present at baseline all lesions up to a maximum of five lesions total (and a maximum of two lesions per organ) representative of all involved organs should be identified as target lesions and will be recorded and measured at baseline (this means in instances where patients have only one or two organ sites involved a maximum of two and four lesions respectively will be recorded). For evidence to support the selection of only five target lesions, see analyses on a large prospective database in the article by Bogaerts et al.

Target lesions should be selected on the basis of their size (lesions with the longest diameter), be representative of all in volved organs, but in addition should be those that lend themselves to reproducible repeated measurements. It may be the case that, on occasion, the largest lesion does not lend itself to reproducible measurement in which circumstance the next largest lesion which can be measured reproducibly should be selected. To illustrate this point see the example in Fig. 3 of Appendix II.

Lymph nodes merit special mention since they are normal anatomical structures which may be visible by imaging even if not involved by tumour. As noted in Section 3, pathological nodes which are defined as measurable and may be identified as target lesions must meet the criterion of a short axis of ≥15 mm by CT scan. Only the short axis of these nodes will contribute to the baseline sum. The short axis of the node is the diameter normally used by radiologists to judge if a node is involved by solid tumour. Nodal size is normally reported as two dimensions in the plane in which the image is obtained (for CT scan this is almost always the axial plane; for MRI the plane of acquisition may be axial, saggital or coronal). The smaller of these measures is the short axis. For example, an abdominal node which is reported as being 20 mm · 30 mm has a short axis of 20 mm and qualifies as a malignant, measurable node. In this example, 20 mm should be recorded as the node measurement (See also the example in Fig. 4 in Appendix II). All other pathological nodes (those with short axis P10 mm but <15 mm) should be considered non-target lesions. Nodes that have a short axis <10 mm are considered non-pathological and should not be recorded or followed.

A sum of the diameters (longest for non-nodal lesions, short axis for nodal lesions) for all target lesions will be calculated and reported as the baseline sum diameters. If lymph nodes are to be included in the sum, then as noted above, only the short axis is added into the sum. The baseline sum diameters will be used as reference to further characterise any objective tumour regression in the measurable dimension of the disease.

All other lesions (or sites of disease) including pathological lymph nodes should be identified as non-target lesions and should also be recorded at baseline. Measurements are not required and these lesions should be followed as ‘present’, ‘absent’, or in rare cases ‘unequivocal progression’ (more details to follow). In addition, it is possible to record multiple nontarget lesions involving the same organ as a single item on the case record form (e.g. ‘multiple enlarged pelvic lymph nodes’ or ‘multiple liver metastases’).

**Response criteria**

This section provides the definitions of the criteria used to determine objective tumour response for target lesions.

**Evaluation of target lesions**

Complete Response (CR): Disappearance of all target lesions. Any pathological lymph nodes (whether target or non-target) must have reduction in short axis to <10 mm.

Partial Response (PR): At least a 30% decrease in the sum of diameters of target lesions, taking as reference the baseline sum diameters.

Progressive Disease (PD): At least a 20% increase in the sum of diameters of target lesions, taking as reference the smallest sum on study (this includes the baseline sum if that is the smallest on study). In addition to the relative increase of 20%, the sum must also demonstrate an absolute increase of at least 5 mm. (Note: the appearance of one or more new lesions is also considered progression).

Stable Disease (SD): Neither sufficient shrinkage to qualify for PR nor sufficient increase to qualify for PD, taking as reference the smallest sum diameters while on study.

**Special notes on the assessment of target lesions**

*Lymph nodes.* Lymph nodes identified as target lesions should always have the actual short axis measurement recorded (measured in the same anatomical plane as the baseline examination), even if the nodes regress to below 10 mm on study. This means that when lymph nodes are included as target lesions, the ‘sum’ of lesions may not be zero even if complete response criteria are met, since a normal lymph node is defined as having a short axis of <10 mm. Case report forms or other data collection methods may therefore be designed to have target nodal lesions recorded in a separate section where, in order to qualify for CR, each node must achieve a short axis <10 mm. For PR, SD and PD, the actual short axis measurement of the nodes is to be included in the sum of target lesions.

*Target lesions that become ‘too small to measure’.* While on study, all lesions (nodal and non-nodal) recorded at baseline should have their actual measurements recorded at each subsequent evaluation, even when very small (e.g. 2 mm). However, sometimes lesions or lymph nodes which are recorded as target lesions at baseline become so faint on CT scan that the radiologist may not feel comfortable assigning an exact measure and may report them as being ‘too small to measure’. When this occurs it is important that a value be recorded on the case report form. If it is the opinion of the radiologist that the lesion has likely disappeared, the measurement should be recorded as 0 mm. If the lesion is believed to be present and is faintly seen but too small to measure, a default value of 5 mm should be assigned (Note: It is less likely that this rule will be used for lymph nodes since they usually have a definable size when normal and are frequently surrounded by fat such as in the retroperitoneum; however, if a lymph node is believed to be present and is faintly seen but too small to measure, a default value of 5 mm should be assigned in this circumstance as well). This default value is derived from the 5 mm CT slice thickness (but should not be changed with varying CT slice thickness). The measurement of these lesions is potentially non-reproducible, therefore providing this default value will prevent false responses or progressions based upon measurement error. To reiterate, however, if the radiologist is able to provide an actual measure, that should be recorded, even if it is below 5 mm.

*Lesions that split or coalesce on treatment.* As noted in Appendix II, when non-nodal lesions ‘fragment’, the longest diameters of the fragmented portions should be added together to calculate the target lesion sum. Similarly, as lesions coalesce, a plane between them may be maintained that would aid in obtaining maximal diameter measurements of each individual lesion. If the lesions have truly coalesced such that they are no longer separable, the vector of the longest diameter in this instance should be the maximal longest diameter for the ‘coalesced lesion’.

**Evaluation of non-target lesions**

This section provides the definitions of the criteria used to determine the tumour response for the group of non-target lesions. While some non-target lesions may actually be measurable, they need not be measured and instead should be assessed only qualitatively at the time points specified in the protocol.

Complete Response (CR): Disappearance of all non-target lesions and normalisation of tumour marker level. All lymph nodes must be non-pathological in size (<10 mm short axis).

Non-CR/Non-PD: Persistence of one or more non-target lesion(s) and/or maintenance of tumour marker level above the normal limits.

Progressive Disease (PD): Unequivocal progression (see comments below) of existing non-target lesions. (Note: the appearance of one or more new lesions is also considered progression).

**Special notes on assessment of progression of non-target disease**

The concept of progression of non-target disease requires additional explanation as follows:

*When the patient also has measurable disease.* In this setting, to achieve ‘unequivocal progression’ on the basis of the non-target disease, there must be an overall level of substantial worsening in non-target disease such that, even in presence of SD or PR in target disease, the overall tumour burden has increased sufficiently to merit discontinuation of therapy (see examples in Appendix II and further details below). A modest ‘increase’ in the size of one or more non-target lesions is usually not sufficient to quality for unequivocal progression status. The designation of overall progression solely on the basis of change in non-target disease in the face of SD or PR of target disease will therefore be extremely rare.

*When the patient has only non-measurable disease.* This circumstance arises in some phase III trials when it is not a criterion of study entry to have measurable disease. The same general concepts apply here as noted above, however, in this instance there is no measurable disease assessment to factor into the interpretation of an increase in non-measurable disease burden.

Because worsening in non-target disease cannot be easily quantified (by definition: if all lesions are truly non-measurable) a useful test that can be applied when assessing patients for unequivocal progression is to consider if the increase in overall disease burden based on the change in non-measurable disease is comparable in magnitude to the increase that would be required to declare PD for measurable disease: i.e. an increase in tumour burden representing an additional 73% increase in‘volume’ (which is equivalent to a 20% increase diameter in a measurable lesion). Examples include an increase in a pleural effusion from ‘trace’ to ‘large’, an increase in lymphangitic disease from localised to widespread, or may be described in protocols as ‘sufficient to require a change in therapy’. Some illustrative examples are shown in Figs. 5 and 6 in Appendix II.If ‘unequivocal progression’ is seen, the patient should be considered to have had overall PD at that point. While it would be ideal to have objective criteria to apply to non-measurable disease, the very nature of that disease makes it impossible to do so, therefore the increase must be substantial.

**New lesions**

The appearance of new malignant lesions denotes disease progression; therefore, some comments on detection of new lesions are important. There are no specific criteria for the identification of new radiographic lesions; however, the finding of a new lesion should be unequivocal: i.e. not attributable to differences in scanning technique, change in imaging modality or findings thought to represent something other than tumour (for example, some ‘new’ bone lesions may be simply healing or flare of pre-existing lesions). This is particularly important when the patient’s baseline lesions show partial or complete response. For example, necrosis of a liver lesion may be reported on a CT scan report as a ‘new’ cystic lesion, which it is not.

A lesion identified on a follow-up study in an anatomical location that was not scanned at baseline is considered a new lesion and will indicate disease progression. An example of this is the patient who has visceral disease at baseline and while on study has a CT or MRI brain ordered which reveals metastases. The patient’s brain metastases are considered to be evidence of PD even if he/she did not have brain imaging at baseline.

If a new lesion is equivocal, for example because of its small size, continued therapy and follow-up evaluation will clarify if it represents truly new disease. If repeat scans con- firm there is definitely a new lesion, then progression should be declared using the date of the initial scan.

While FDG-PET response assessments need additional study, it is sometimes reasonable to incorporate the use of FDG-PET scanning to complement CT scanning in assessment of progression (particularly possible ‘new’ disease). New lesions on the basis of FDG-PET imaging can be identified according to the following algorithm:

a. Negative FDG-PET at baseline, with a positive FDG-PET at follow-up is a sign of PD based on a new lesion.

b. No FDG-PET at baseline and a positive FDG-PET at follow-up: If the positive FDG-PET at follow-up corresponds to a new site of disease confirmed by CT, this is PD. If the positive FDG-PET at follow-up is not confirmed as a new site of disease on CT, additional follow-up CT scans are needed to determine if there is truly progression occurring at that site (if so, the date of PD will be the date of the initial abnormal FDG-PET scan). If the positive FDG-PET at follow-up corresponds to a pre-existing site of disease on CT that is not progressing on the basis of the anatomic images, this is not PD.

**Evaluation of best overall response**

The best overall response is the best response recorded from the start of the study treatment until the end of treatment taking into account any requirement for confirmation. On occasion a response may not be documented until after the end of therapy so protocols should be clear if post-treatment assessments are to be considered in determination of best overall response. Protocols must specify how any new therapy introduced before progression will affect best response designation. The patient’s best overall response assignment will depend on the findings of both target and non-target disease and will also take into consideration the appearance of new lesions. Furthermore, depending on the nature of the study and the protocol requirements, it may also require confirmatory measurement (see Section 4.6). Specifically, in non-randomised trials where response is the primary endpoint, confirmation of PR or CR is needed to deem either one the ‘best overall response’. This is described further below.

**Time point response**

It is assumed that at each protocol specified time point, a response assessment occurs. Table 1 on the next page provides a summary of the overall response status calculation at each time point for patients who have measurable disease at baseline.

When patients have non-measurable (therefore non-target) disease only, Table 2 is to be used.

**Missing assessments and inevaluable designation**

When no imaging/measurement is done at all at a particular time point, the patient is not evaluable (NE) at that time point. If only a subset of lesion measurements are made at an assessment, usually the case is also considered NE at that time point, unless a convincing argument can be made that the contribution of the individual missing lesion(s) would not change the assigned time point response. This would be most likely to happen in the case of PD. For example, if a patient had a baseline sum of 50 mm with three measured lesions and at follow-up only two lesions were assessed, but those gave a sum of 80 mm, the patient will have achieved PD status, regardless of the contribution of the missing lesion.

**Best overall response: all time points**

The best overall response is determined once all the data for the patient is known.

Best response determination in trials where confirmation of complete or partial response IS NOT required: Best response in these trials is defined as the best response across all time points (for example, a patient who has SD at first assessment, PR at second assessment, and PD on last assessment has a best overall response of PR). When SD is believed to be best response, it must also meet the protocol specified minimum time from baseline. If the minimum time is not met when SD is otherwise the best time point response, the patient’s best response depends on the subsequent assessments. For example, a patient who has SD at first assessment, PD at second and does not meet minimum duration for SD, will have a best response of PD. The same patient lost to follow-up after the first SD assessment would be considered inevaluable.

Best response determination in trials where confirmation of complete or partial response IS required: Complete or partial responses may be claimed only if the criteria for each are met at a subsequent time point as specified in the protocol (generally 4 weeks later). In this circumstance, the best overall response can be interpreted as in Table 3.

Table 1 Time point response: patients with target (+/– non-target) disease

| **Target lesions** | **Non-target lesions** | **New lesions** | **Overall response** |
| --- | --- | --- | --- |
| CR | CR | No | CR |
| CR | Non-CR/non-PD | No | PR |
| CR | Not evaluated | No | PR |
| PR | Non-PD or not all evaluated | No | PR |
| SD | Non-PD or not all evaluated | No | SD |
| Not all evaluated | Non-PD | No | NE |
| PD  Any  Any | Any  PD  Any | Yes or No  Yes or No  Yes | PD  PD  PD |

Note: CR=complete response, PR=partial response, SD=stable disease, PD=progressive disease, NE=inevaluable.

Table 2 Time point response: patients with non-target disease only

| **Non-target lesions** | **New lesions** | **Overall response** |
| --- | --- | --- |
| CR | No | CR |
| Non-CR/non-PD | No | Non-CR/non-PD |
| Not all evaluated | No | NE |
| Unequivocal PD | Yes or No | PD |
| Any | Yes | PD |

Note: CR=complete response, PD=progressive disease, NE=inevaluable.
‘Non-CR/non-PD’ is preferred over ‘stable disease’ for non-target disease since SD is increasingly used as endpoint for assessment of efficacy in some trials so to assign this category when no lesions can be measured is not advised.

Table 3 Best overall response when confirmation of CR and PR required

| **Overall response First time point** | **Overall response Subsequent time point** | **BEST overall response** |
| --- | --- | --- |
| CR | CR | CR |
| CR | PR | SD, PD, or PR^a^ |
| CR | SD | SD provided minimum criteria for SD duration met, otherwise, PD |
| CR | PD | SD provided minimum criteria for SD duration met, otherwise, PD |
| CR | NE | SD provided minimum criteria for SD duration met, otherwise NE |
| PR | CR | PR |
| PR | PR | PR |
| PR | SD | SD |
| PR | PD | SD provided minimum criteria for SD duration met, otherwise, PD |
| PR | NE | SD provided minimum criteria for SD duration met, otherwise NE |
| NE | NE | NE |

Note: CR=complete response, PR=partial response, SD=stable disease, PD=progressive disease, NE=inevaluable.

a. If a CR is truly met at first time point, then any disease seen at a subsequent time point, even disease meeting PR criteria relative to baseline, makes the disease PD at that point (since disease must have reappeared after CR). Best response would depend on whether minimum duration for SD was met. However, sometimes ‘CR’ may be claimed when subsequent scans suggest small lesions were likely still present and in fact the patient had PR, not CR at the first time point. Under these circumstances, the original CR should be changed to PR and the best response is PR.

**Special notes on response assessment**

When nodal disease is included in the sum of target lesions and the nodes decrease to ‘normal’ size (<10 mm), they may still have a measurement reported on scans. This measurement should be recorded even though the nodes are normal in order not to overstate progression should it be based on increase in size of the nodes. As noted earlier, this means that patients with CR may not have a total sum of ‘zero’ on the case report form (CRF).

In trials where confirmation of response is required, repeated ‘NE’ time point assessments may complicate best response determination. The analysis plan for the trial must address how missing data/assessments will be addressed in determination of response and progression. For example, in most trials it is reasonable to consider a patient with time point responses of PR-NE-PR as a confirmed response.

Patients with a global deterioration of health status requiring discontinuation of treatment without objective evidence of disease progression at that time should be reported as‘symptomatic deterioration’. Every effort should be made to document objective progression even after discontinuation of treatment. Symptomatic deterioration is not a descriptor of an objective response: it is a reason for stopping study therapy. The objective response status of such patients is to be determined by evaluation of target and non-target disease as shown in Tables 1–3.

Conditions that define ‘early progression, early death and inevaluability’ are study specific and should be clearly described in each protocol (depending on treatment duration, treatment periodicity).

In some circumstances it may be difficult to distinguish residual disease from normal tissue. When the evaluation of complete response depends upon this determination, it is recommended that the residual lesion be investigated (fine needle aspirate/biopsy) before assigning a status of complete response. FDG-PET may be used to upgrade a response to a CR in a manner similar to a biopsy in cases where a residual radiographic abnormality is thought to represent fibrosis or scarring. The use of FDG-PET in this circumstance should be prospectively described in the protocol and supported by disease specific medical literature for the indication. However, it must be acknowledged that both approaches may lead to false positive CR due to limitations of FDG-PET and biopsy resolution/sensitivity. For equivocal findings of progression (e.g. very small and uncertain new lesions; cystic changes or necrosis in existing lesions), treatment may continue until the next scheduled assessment. If at the next scheduled assessment, progression is confirmed, the date of progression should be the earlier date when progression was suspected.

**Frequency of tumour re-evaluation**

Frequency of tumour re-evaluation while on treatment should be protocol specific and adapted to the type and schedule of treatment. However, in the context of phase II studies where the beneficial effect of therapy is not known, follow-up every 6–8 weeks (timed to coincide with the end of a cycle) is reasonable. Smaller or greater time intervals than these could be justified in specific regimens or circumstances. The protocol should specify which organ sites are to be evaluated at baseline (usually those most likely to be involved with metastatic disease for the tumour type under study) and how often evaluations are repeated. Normally, all target and non-target sites are evaluated at each assessment. In selected circumstances certain non-target organs may be evaluated less frequently. For example, bone scans may need to be repeated only when complete response is identified in target disease when progression in bone is suspected.

After the end of the treatment, the need for repetitive tumour evaluations depends on whether the trial has as a goal the response rate or the time to an event (progression/death). If ‘time to an event’ (e.g. time to progression, disease-free survival, progression-free survival) is the main endpoint of the study, then routine scheduled re-evaluation of protocol specified sites of disease is warranted. In randomised comparative trials in particular, the scheduled assessments should be performed as identified on a calendar schedule (for example: every 6–8 weeks on treatment or every 3–4 months after treatment) and should not be affected by delays in therapy, drug holidays or any other events that might lead to imbalance in a treatment arm in the timing of disease assessment.

**Confirmatory measurement/duration of response**

**Confirmation**

In non-randomised trials where response is the primary endpoint, confirmation of PR and CR is required to ensure responses identified are not the result of measurement error.

This will also permit appropriate interpretation of results in the context of historical data where response has traditionally required confirmation in such trials (see the paper by Bogaerts

et al. in this Special Issue). However, in all other circumstances, i.e. in randomised trials (phase II or III) or studies where stable disease or progression are the primary endpoints, confirmation of response is not required since it will not add value to the interpretation of trial results. However, elimination of the requirement for response confirmation may increase the importance of central review to protect against bias, in particular in studies which are not blinded.

In the case of SD, measurements must have met the SD criteria at least once after study entry at a minimum interval (in general not less than 6–8 weeks) that is defined in the study protocol.

**Duration of overall response**

The duration of overall response is measured from the time measurement criteria are first met for CR/PR (whichever is first recorded) until the first date that recurrent or progressive disease is objectively documented (taking as reference for progressive disease the smallest measurements recorded on study).

The duration of overall complete response is measured from the time measurement criteria are first met for CR until the first date that recurrent disease is objectively documented.

**Duration of stable disease**

Stable disease is measured from the start of the treatment (in randomised trials, from date of randomisation) until the criteria for progression are met, taking as reference the smallest sum on study (if the baseline sum is the smallest, this is the reference for calculation of PD).

The clinical relevance of the duration of stable disease varies in different studies and diseases. If the proportion of patients achieving stable disease for a minimum period of time is an endpoint of importance in a particular trial, the protocol should specify the minimal time interval required between two measurements for determination of stable disease.

Note: The duration of response and stable disease as well as the progression-free survival are influenced by the frequency of follow-up after baseline evaluation. It is not in the scope of this guideline to define a standard follow-up frequency. The frequency should take into account many parameters including disease types and stages, treatment periodicity and standard practice. However, these limitations of the precision of the measured endpoint should be taken into account if comparisons between trials are to be made.

**Progression-free survival/proportion progression-free**

**Phase II trials**

This guideline is focused primarily on the use of objective response endpoints for phase II trials. In some circumstances, ‘response rate’ may not be the optimal method to assess the potential anticancer activity of new agents/regimens. In such cases ‘progression-free survival’ (PFS) or the ‘proportion progression-free’ at landmark time points, might be considered appropriate alternatives to provide an initial signal of biologic effect of new agents. It is clear, however, that in an uncontrolled trial, these measures are subject to criticism since an apparently promising observation may be related to biological factors such as patient selection and not the impact of the intervention.

Thus, phase II screening trials utilizing these endpoints are best designed with a randomised control. Exceptions may exist where the behaviour patterns of certain cancers are so consistent (and usually consistently poor), that a non-randomised trial is justifiable (see for example van Glabbeke et al.). However, in these cases it will be essential to document with care the basis for estimating the expected PFS or proportion progression-free in the absence of a treatment effect.

**Phase III trials**

Phase III trials in advanced cancers are increasingly designed to evaluate progression-free survival or time to progression as the primary outcome of interest. Assessment of progression is relatively straightforward if the protocol requires all patients to have measurable disease. However, restricting entry to this subset of patients is subject to criticism: it may result in a trial where the results are less likely to be generalisable if, in the disease under study, a substantial proportion of patients would be excluded. Moreover, the restriction to entry will slow recruitment to the study. Increasingly, therefore, trials allow entry of both patients with measurable disease as well as those with non-measurable disease only. In this circumstance, care must be taken to explicitly describe the findings which would qualify for progressive disease for those patients without measurable lesions. Furthermore, in this setting, protocols must indicate if the maximum number of recorded target lesions for those patients with measurable disease may be relaxed from five to three (based on the data found in Bogaerts et.al. and Moskowitz et al.). As found in the ‘special notes on assessment of progression’, these guidelines offer recommendations for assessment of progression in this setting. Furthermore, if available, validated tumour marker measures of progression (as has been proposed for ovarian cancer) may be useful to integrate into the definition of progression. Centralized blinded review of imaging studies or of source imaging reports to verify ‘unequivocal progression’ may be needed if important drug development or drug approval decisions are to be based on the study outcome. Finally, as noted earlier, because the date of progression is subject to as certainment bias, timing of investigations in study arms should be the same. The article by Dancey et al. in this special issue provides a more detailed discussion of the assessment of progression in randomised trials.

**Independent review of response and progression**

For trials where objective response (CR + PR) is the primary endpoint, and in particular where key drug development decisions are based on the observation of a minimum number of responders, it is recommended that all claimed responses be reviewed by an expert(s) independent of the study. If the study is a randomised trial, ideally reviewers should be blinded to treatment assignment. Simultaneous review of the patients’ files and radiological images is the best approach.

Independent review of progression presents some more complex issues: for example, there are statistical problems with the use of central-review-based progression time in place of investigator-based progression time due to the potential introduction of informative censoring when the former precedes the latter. An overview of these factors and other lessons learned from independent review is provided in an article by Ford et al. in this special issue

**Reporting best response results**

**Phase II trials**

When response is the primary endpoint, and thus all patients must have measurable disease to enter the trial, all patients included in the study must be accounted for in the report of the results, even if there are major protocol treatment deviations or if they are not evaluable. Each patient will be assigned one of the following categories:

1. Complete response

2. Partial response

3. Stable disease

4. Progression

5. Inevaluable for response: specify reasons (for example: early death, malignant disease; early death, toxicity; tumour assessments not repeated/incomplete; other (specify)).

Normally, all eligible patients should be included in the denominator for the calculation of the response rate for phase II trials (in some protocols it will be appropriate to include all treated patients). It is generally preferred that 95% two-sided confidence limits are given for the calculated response rate. Trial conclusions should be based on the response rate for all eligible (or all treated) patients and should not be based on a selected ‘evaluable’ subset.

**Phase III trials**

Response evaluation in phase III trials may be an indicator of the relative anti-tumour activity of the treatments evaluated and is almost always a secondary endpoint. Observed differences in response rate may not predict the clinically relevant therapeutic benefit for the population studied. If objective response is selected as a primary endpoint for a phase III study (only in circumstances where a direct relationship between objective tumour response and a clinically relevant therapeutic benefit can be unambiguously demonstrated for the population studied), the same criteria as those applying to phase II trials should be used and all patients entered should have at least one measurable lesion.

In those many cases where response is a secondary endpoint and not all trial patients have measurable disease, the method for reporting overall best response rates must be pre-specified in the protocol. In practice, response rate may be reported using either an ‘intent to treat’ analysis (all randomised patients in the denominator) or an analysis where only the subset of patients with measurable disease at baseline are included. The protocol should clearly specify how response results will be reported, including any subset analyses that are planned.

The original version of RECIST suggested that in phase III trials one could write protocols using a ‘relaxed’ interpretation of the RECIST guidelines (for example, reducing the number of lesions measured) but this should no longer be done since these revised guidelines have been amended in such a way that it is clear how these criteria should be applied for all trials in which anatomical assessment of tumour response or progression are endpoints.

# Attachment 2: Performance status (ECOG scale)

| ECOG  GRADE | DESCRIPTION |
| --- | --- |
| 0 | Fully active, able to carry on all pre-disease performance without restriction. |
| 1 | Restricted in physically strenuous activity, but ambulatory and able to carry out work of a light or sedentary nature, i.e. light housework, office work |
| 2 | Ambulatory and capable of self-care, but unable to carry out any work activities. Up and about more than 50% of waking hours. |
| 3 | Capable of only limited self-care, confine to bed or chair more than 50% of waking hours |
| 4 | Completely disabled. Cannot carry on any self-care. Totally confined to bed or chair. |
